# Hafnium(IV) Chemistry with Imide–Dioxime and Catecholate–Oxime Ligands: Unique {Hf_5_} and Metalloaromatic {Hf_6_}–Oxo Clusters Exhibiting Fluorescence

**DOI:** 10.1021/acs.inorgchem.2c01768

**Published:** 2022-12-03

**Authors:** Stamatis S. Passadis, Sofia Hadjithoma, Nicola J. Fairbairn, Gordon J. Hedley, Nuno A. G. Bandeira, Athanassios C. Tsipis, Haralampos N. Miras, Anastasios D. Keramidas, Themistoklis A. Kabanos

**Affiliations:** † Section of Inorganic and Analytical Chemistry, University of Ioannina, Ioannina45110, Greece; ‡ Department of Chemistry, University of Cyprus, Nicosia1678, Cyprus; § WestCHEM, School of Chemistry, University of Glasgow, GlasgowG12 8QQ, U.K.; ∥ BioISI—BioSystems and Integrative Sciences Institute, Faculdade de Ciências da Universidade de Lisboa, Campo Grande, 1749-016Lisboa, Portugal

## Abstract

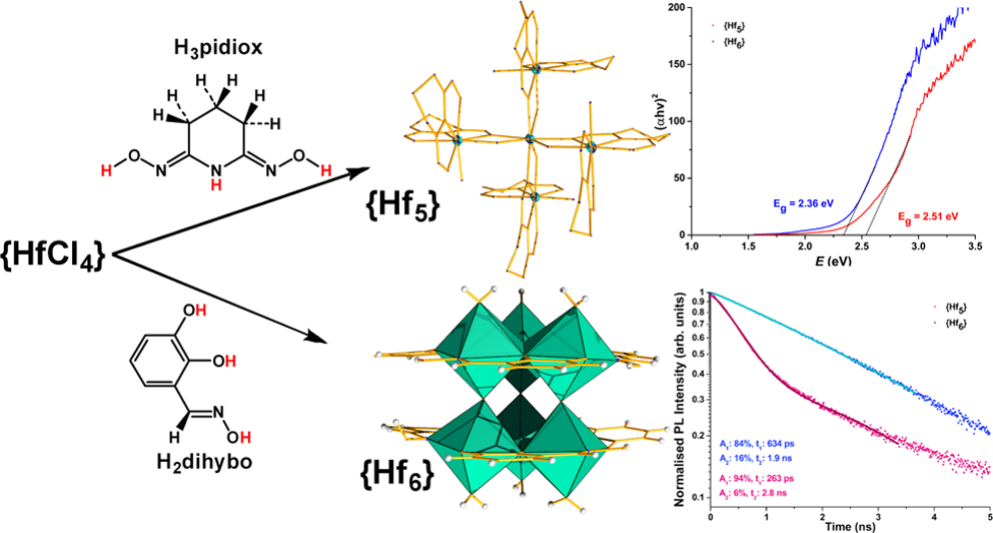

Hafnium(IV) molecular species have gained increasing attention due to their numerous applications ranging from high-resolution nanolithography, heterogeneous catalysis, and electronics to the design of molecule-based building blocks in metal–organic frameworks (MOFs), with applications in gas separation, sorption, luminescence sensing, and interim storage of radioactive waste. Despite great potential, their chemistry is relatively underdeveloped. Here, we use strong chelators (2*Z*-6*Z*)-piperidine-2,6-dione (H_3_pidiox) and 2,3-dihydroxybenzaldehyde oxime (H_3_dihybo) to synthesize the first ever reported pentanuclear {Hf_5_/H_3_pidiox} and hexanuclear {Hf_6_/H_3_dihybo} clusters (HfOCs). The {Hf_6_} clusters adopt unique core structures [Hf_6_^IV^(μ_3_-O)_2_(μ-O)_3_] with a trigonal-prismatic arrangement of the six hafnium atoms and have been characterized via single-crystal X-ray diffraction analysis, UV–vis spectroscopy in the solid state, NMR, fluorescence spectroscopy, and high-resolution mass spectrometry in solution. One-dimensional (1D) and two-dimensional (2D) ^1^H NMR and mass spectroscopies reveal the exceptional thermodynamic stability of the HfOCs in solution. Interestingly, the conjunction of the oxime group with the catechol resulted in the remarkable reduction of the clusters’ band gap, below 2.51 eV. Another prominent feature is the occurrence of pronounced metalloaromaticity of the triangular {Hf_3_} metallic component revealed by its NICS*_zz_* scan curve calculated by means of density functional theory (DFT). The NICS*_zz_*(1) value of −44.6 ppm is considerably higher than the −29.7 ppm found at the same level of theory for the benzene ring. Finally, we investigated the luminescence properties of the clusters where **1** emits light in the violet region despite the lack of fluorescence of the free H_3_pidiox ligand, whereas the {Hf_6_} **3** shifts the violet-emitting light of the H_3_dihybo to lower energy. DFT calculations show that this fluorescence behavior stems from ligand-centered molecular orbital transitions and that Hf^IV^ coordination has a modulating effect on the photophysics of these HfOCs. This work not only represents a significant milestone in the construction of stable low-band-gap multinuclear Hf^IV^ clusters with unique structural features and metal-centered aromaticity but also reveals the potential of Hf(IV) molecule-based materials with applications in sensing, catalysis, and electronic devices.

## Abstract 2

The reaction of HfCl_4_ with dioxime/amine (H_3_pidiox) and the catechol/oxime (H_3_dihybo) organic chelators gave the first pentanuclear and the first metalloaromatic hexanuclear {Hf_6_} cluster. The combined effect of the oxime and catechol groups resulted in the remarkable reduction of the {Hf_6_} cluster’s band gap, below 2.51 eV and induced fluorescence in solution at room temperature, making these clusters highly promising candidates for applications in sensing and optoelectronics.

## Introduction

Group IV metal oxo clusters (MOCs) are polynuclear compounds exhibiting an inorganic core formed by group IV metals in their highest oxidation state linked by oxygen atoms and stabilized by capping ligands. Although there are many reported applications of group IV MOCs,^1−8^ their chemistry is still underdeveloped^9−13^ and particularly the hafnium chemistry in marked contrast to the titanium and zirconium. HfOCs have potential applications in high-resolution nanolithography,^14−16^ in heterogeneous catalysis,^17^ and they have been used as molecule-based building blocks in metal–organic frameworks (MOFs), with applications in gas separation,^18,19^ sorption,^20^ catalysis,^21−28^ luminescence sensing,^29^ and interim storage of radioactive waste.^30^

In addition, the study of HfOCs is of fundamental importance because they are processable molecular analogues of HfO_2_ which have numerous applications including protective surface coatings,^31^ metal-oxide-semiconductor field-effect transistors,^32,33^ random access memory devices,^34−37^ and teeth prosthetics.^38^

The band gap energy of HfO_2_ ranges from 5.3 to 6.0 eV depending on its different phases and its formation under different experimental conditions.^39^ This large band gap is practically prohibitive for the utilization of HfO_2_ in photocatalytic applications. The modulation of the band gap can be achieved by employing organic chelators^40−42^ leading to visible light absorption by HfOCs.

**Scheme 1:**
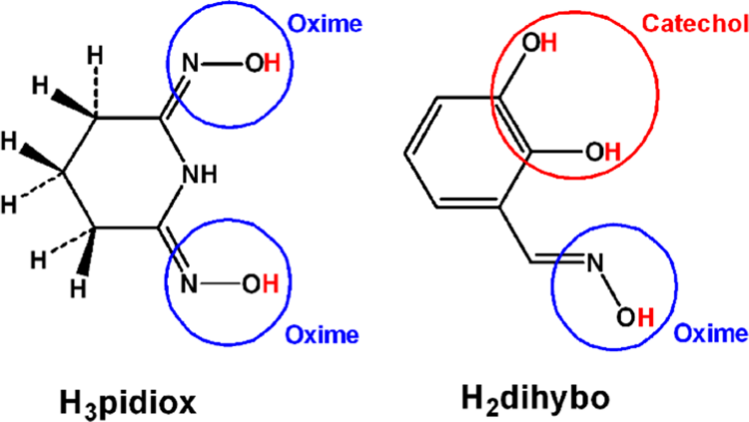
Ligands H_3_pidiox and H_3_dihybo

Recently, our group studied the reaction of MCl_4_ (M = Ti^IV^, Zr^IV^) with the organic ligands (2*Z*,6*Z*)-piperidine-2,6-dione dioxime (H_3_pidiox)^43,44^ and 2,3-dihydroxybenzaldehyde (H_3_dihybo) (Scheme 1).^40^ Both ligands are strong binders to hard metals in their highest oxidation state,^45,46^ and have been used for the extraction of heavy hard metal ions from seawater.^47−49^ The employment of H_3_pidiox and H_3_dihybo led to the formation of polynuclear clusters with unique structural features, allowing the induction of metalloaromaticity and the modulation of the compounds’ band gap. The reaction of HfCl_4_ with the ligand H_3_dihybo gave a hexanuclear cluster, {Hf_6_}, with two cyclo-{Hf_3_} metallic cores which exhibit metalloaromaticity.

The discovery of the aromatic nature of benzene had a great impact on many fields of science such as organic, industrial, and medicinal chemistry, and life sciences.^50^ The extensive investigation of metalloaromaticity that took place in the last few years, led to a better understanding of the concept and exponential growth of examples in the literature^51^ due to the discovery of new metalloaromatic species such as metallo-benzenes^52^ and all-metal clusters [i.e., (Al_4_^2–^),^53^ (Au_5_Zn^+^)^54^]. Owing to the clusters’ metalloaromaticity-induced wide range of applications^50,55−57^ such as catalysis, drugs, molecular electronic devices, aerospace engineering, molecular magnets, the revelation of unknown aspects of chemical bonding, and understanding the electronic and surface properties of metal oxides, and mixed-metal clusters, this family of compounds has attracted the attention of numerous research groups. Metalloaromaticity of all-metal cyclic species is due to the formation of σ, π, δ, and φ molecular orbitals (MOs),^50^ in marked contrast to the organic aromatic rings where electron delocalization is supported by π MOs only.^50^ Manifestation of metalloaromatic behavior has been evaluated using several different criteria that have been developed over the years that are directly related to structural, energetic, magnetic, and electronic characteristics of the reported species. Among them, the magnetic nucleus-independent chemical shift (NICS) criterion appears to be one of the most powerful.^58^ Therefore, in this work, we employ the NICS criterion to investigate whether the cyclic trinuclear {Hf_3_} metallic ring cores of the newly synthesized compounds exhibit metalloaromatic behavior.

Based on the above observations, our work is inspired by the prospect of generating new building units for the construction of metal–organic frameworks with potential application in catalysis, sensing, and gas separation. Moreover, the combination of the inherent inertness of Hf species and higher stabilities compared to first-row transition metals along with the highly polarized Hf–X bonds will potentially influence the catalytic, optoelectronic, and luminescence sensing applications. Herein, we report the synthesis, structure, and physicochemical characterization of the pentanuclear HfOC [Hf_5_(μ-OH)_4_(OH_2_)_4_(μ-η^1^,η^1^,η^2^-Hpidiox-*O*,*N*,*O*′)_4_(η^1^,η^1^,η^1^-Hpidiox-*O*,*N*,*O*′)_4_]·KCl·3.25CH_3_OH·16.5H_2_O (**1**) and of three hexanuclear HfOCs with general formula [Hf_6_(μ_3_-O)_2_(μ-O)_3_(OH_2_)_6_(μ-η^1^,η^2^,η^1^-Hdihybo-*O*,*O*′,*N*)_6_] (**2**–**4**) with the organic chelators H_3_pidiox and H_3_dihybo. HfOC **1** is the first example of a pentanuclear hafnium cluster to be reported.^9^ Both the core structure [Hf_6_(μ_3_-O)_2_(μ-O)_3_] and the trigonal-prismatic arrangement of the six hafnium atoms in compounds **2**–**4** are unique. Moreover, the ligation of H_3_pidiox and H_3_dihybo ligands to Hf^IV^ induces fluorescence in solution at room temperature, rendering these Hf/H_3_pidiox/H_3_dihybo clusters highly promising candidates for applications in sensing, catalysis, and optoelectronics.^59−62^

## Experimental Section

### Synthesis of the HfOCs **1**–**4**

#### Experimental Details

All chemicals and solvents purchased from Sigma-Aldrich and Merck were of reagent grade and used without further purification. C, H, and N analyses were conducted by the microanalytical service of the School of Chemistry, the University of Glasgow. Samples for EA were collected after the removal of the single crystals from the mother liquor by filtration and dried on the bench for 2 days.

##### Synthesis of [Hf_5_(μ-OH)_4_(OH_2_)_4_(μ-η^1^,η^1^,η^2^-Hpidiox-*O*,*N*,*O*′)_4_(η^1^,η^1^,η^1^-Hpidiox-*O*,*N*,*O*′)_4_]·KCl·3CH_3_OH·16H_2_O (**1**)

To a stirred moist methyl alcohol solution (4 mL) were successively added H_3_pidiox (89.3 mg, 0.624 mmol) and HfCl_4_ (100 mg, 0.312 mmol). Then, upon addition of one portion of solid KOH (35 mg, 0.624 mmol), a white precipitate was formed which was filtered off and the colorless filtrate was kept at room temperature (∼20 °C) for 5–6 days, during which period white crystals were formed. The crystals were filtered to obtain 98.6 mg of HfOC **1**. (Yield: 60%, based on HfCl_4_.) Anal. Calcd for (C_40_H_92_N_24_O_24_Hf_5_·KCl·3CH_3_OH·16H_2_O, *M*_r_ = 2602.42 g mol^–1^): C 19.85, H 3.64, N 12.92; found: C 19.63, H 4.31, N 12.72.

##### Synthesis of [Hf_6_^IV^(μ_3_-O)_2_(μ-O)_3_(OH_2_)_6_(μ-η^1^,η^2^,η^1^-Hdihybo-*O*,*O*′,*N*)_6_]Cl_2_·2Bu_4_NCl·2CH_3_OH·4H_2_O (**2**)

To a stirred moist methyl alcohol solution (4 mL) were successively added H_3_dihybo (47.8 mg, 0.312 mmol) and HfCl_4_ (100.0 mg, 0.312 mmol). The colorless solution of the ligand turned light orange upon addition of HfCl_4_. Then, 1.6 mL of tetrabutylammonium hydroxide, 0.39 M in methyl alcohol (162.0 mg, 0.624 mmol) was added in one portion. The solution was filtered and a light orange filtrate was obtained and was kept at room temperature (∼20 °C) for 4–5 days, during which period yellow crystals were formed. We were unable to prepare an analytically pure sample on a preparative scale because the compound is very hygroscopic. Even though single crystals were also obtained in this case, the data were not of publishable quality due to the hygroscopic nature of the single crystal but allowed us to determine the content of the unit cell (see Figure S1) and provide the formula above.

##### [Hf_6_^IV^(μ_3_-O)_2_(μ-O)_3_(OH_2_)_6_(μ-η^1^,η^2^,η^1^-Hdihybo-*O*,*O*′,*N*)_6_]Cl_2_·(Et_3_NHCl)·2CH_3_OH·1.5H_2_O (**3**)

To a stirred moist methyl alcohol solution (4 mL) were successively added H_3_dihybo (47.8 mg, 0.312 mmol) and HfCl_4_ (100.0 mg, 0.312 mmol). The colorless solution of the ligand turned light orange upon addition of HfCl_4_. Then, triethylamine (0.087 mL, 0.624 mmol) was added in one portion. The solution was stirred for 5 min, filtered, and the light orange filtrate was obtained and was kept at room temperature (∼20 °C) for 4–5 days, during which period yellow crystals were formed. The crystals were filtered to obtain 103 mg of HfOC **3**. (Yield: 82%, based on HfCl_4_.) Anal. Calcd for ((C_42_H_48_N_6_O_29_Hf_6_Cl_2_·(Et_3_NHCl)·2CH_3_OH·1.5H_2_O), *M*_r_ = 2470.355 g mol^–1^): C 24.31, H 2.69, N 3.97; found: C 23.85, H 2.70, N 4.02.

##### {[Hf_6_^IV^(μ_3_-O)_2_(μ-O)_3_(OCH_3_)_2_(OH_2_)_4_(μ-η^1^,η^2^,η^1^-Hdihybo-*O*,*O*′,*N*)_6_][Hf_6_^IV^(μ_3_-O)_2_(μ-O)_3_(OH_2_)_6_(μ-η^1^,η^2^,η^1^-Hdihybo-*O*,*O*′,*N*)_6_]}Cl_2_·C_5_H_5_N·CH_3_OH·5H_2_O (**4**)

To a stirred moist methyl alcohol solution (4 mL) were successively added H_3_dihybo (47.8 mg, 0.312 mmol) and HfCl_4_ (100.0 mg, 0.312 mmol). The colorless solution of the ligand turned light orange upon addition of HfCl_4_. Then, pyridine (0.05 mL, 0.624 mmol) was added in one portion. The solution was stirred for 5 min, filtered, and the light orange filtrate was kept at room temperature (∼20 °C) for 4–5 days, during which period yellow crystals were formed. The crystals were filtered to obtain 93 mg of HfOC **4**. (Yield: 75%, based on HfCl_4_). Anal. Calcd for (C_42_H_42_N_6_O_29_Hf_6_Cl_2_·C_5_H_5_N·CH_3_OH·5H_2_O, *M*_r_ = 2449.88 g mol^–1^): C 24.02, H 2.51, N 4.00; found: C 23.81, H 2.49, N 4.07.

### Computational Details

The Amsterdam Density Functional^63^ (ADF 2019.304) program suite was used for the calculation of excited state geometries. The revised^64^ Perdew, Burke, and Ernzerhof^65^ gradient corrected density functional with Grimme’s fourth generation dispersion correction^66^ (revPBE-D4) was used together with a Slater type basis set of triple zeta quality augmented with an additional polarization function (TZP). The COSMO^67^ implicit solvation scheme was also employed with the default parameters for methanol. Due to the presence of heavy elements, the scalar Zero Order Regular Approximated (ZORA) Hamiltonian^68^ was applied in the optimization runs. Geometry optimizations were performed on the {Hf_5_} and {Hf_6_} molecular models constrained to the *C*_2_ point group symmetry to allow for Jahn–Teller distortions in the excited state. Vertical excited state energies were computed with the spin–orbit perturbative approach (SOPERT) of the time-dependent^69^ density functional (TD-revPBE-D4) and these showed no changes in either transition energy or oscillator strengths relative to the spin-free wavefunctions. We found that this approach affords a reasonable compromise between accuracy and computational performance.

The calculation of the NICS values employed the gauge-including atomic orbital (GIAO) DFT method^70,71^ as implemented in the Gaussian09 series of programs^72^ employing the PBE0 functional in combination with the 6-31G(d,p) basis set for all nonmetal atoms, E and the Def2-TZVP basis set for Hf atoms (the computational protocol is denoted as GIAO/PBE0/Def2-TZVP(Hf)U6-31G(d,p)(E)).

## Results and Discussion

### Synthesis of the HfOCs **1**–**4**

**Scheme 2:**
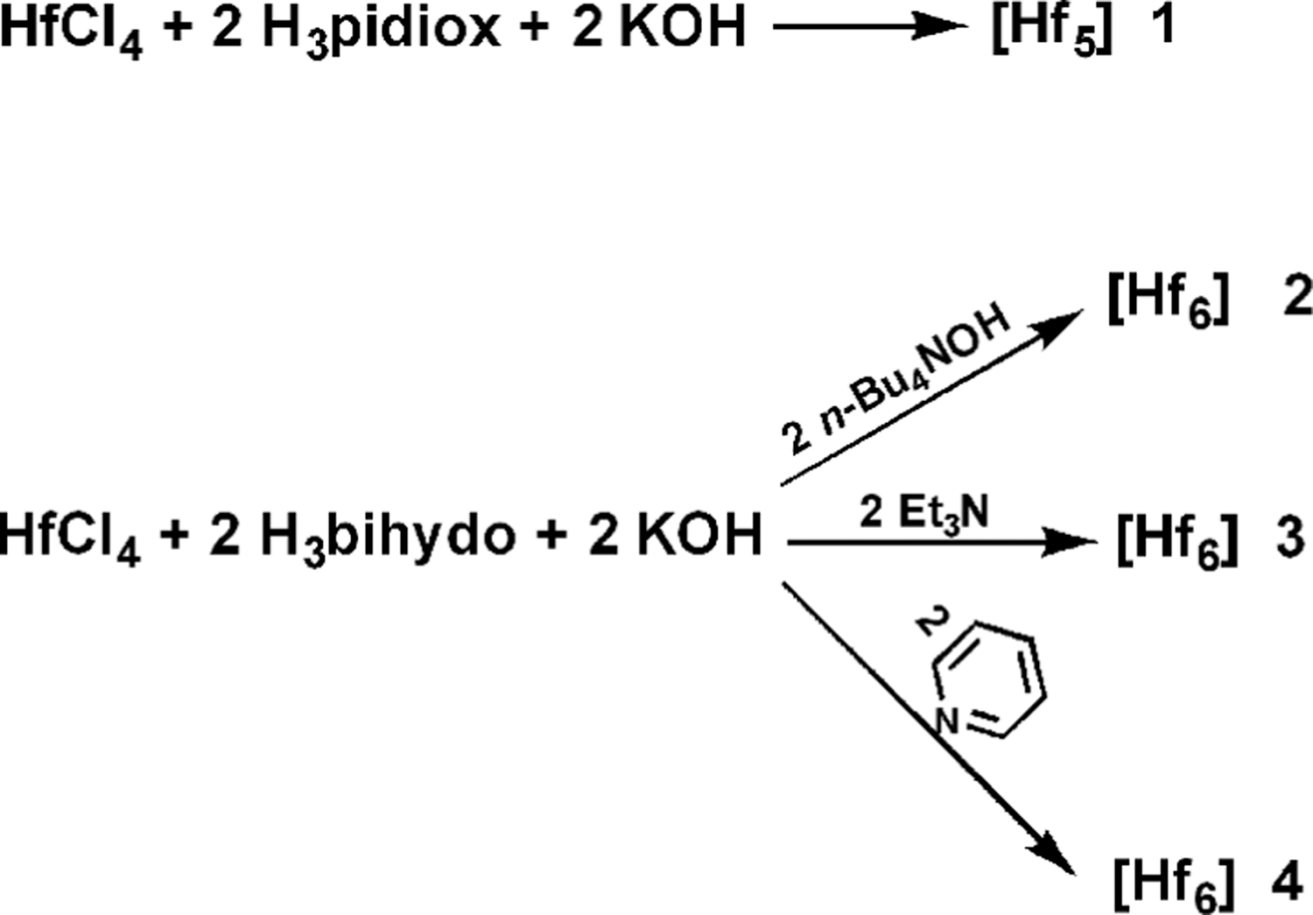
Synthesis of HfOCs **1** {Hf_5_}, **2**, **3**, and **4** {Hf_6_}

Pentanuclear HfOC/H_3_pidiox **1** was synthesized via a one-pot three-component reaction (eq 1 and Scheme 2) at room temperature, while the hexanuclear HfOCs/H_3_dihybo **2**, **3**, and **4** (eq 2 and Scheme 2) were also synthesized in a similar fashion. When KOH was used as a base in the reaction mixture of HfCl_4_ with H_3_dihybo, no suitable crystals for X-ray structure analysis of {Hf_6_} were obtained, while the use of ^*n*^Bu_4_NOH led to hygroscopic {Hf_6_} **2**. Finally, the use of Et_3_N and pyridine resulted in the isolation of {Hf_6_} HfOCs **3** and **4**, respectively, which were suitable for physicochemical measurements.
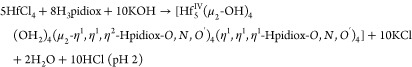
1
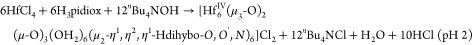
2

### Crystal Structures

**Figure 1:**
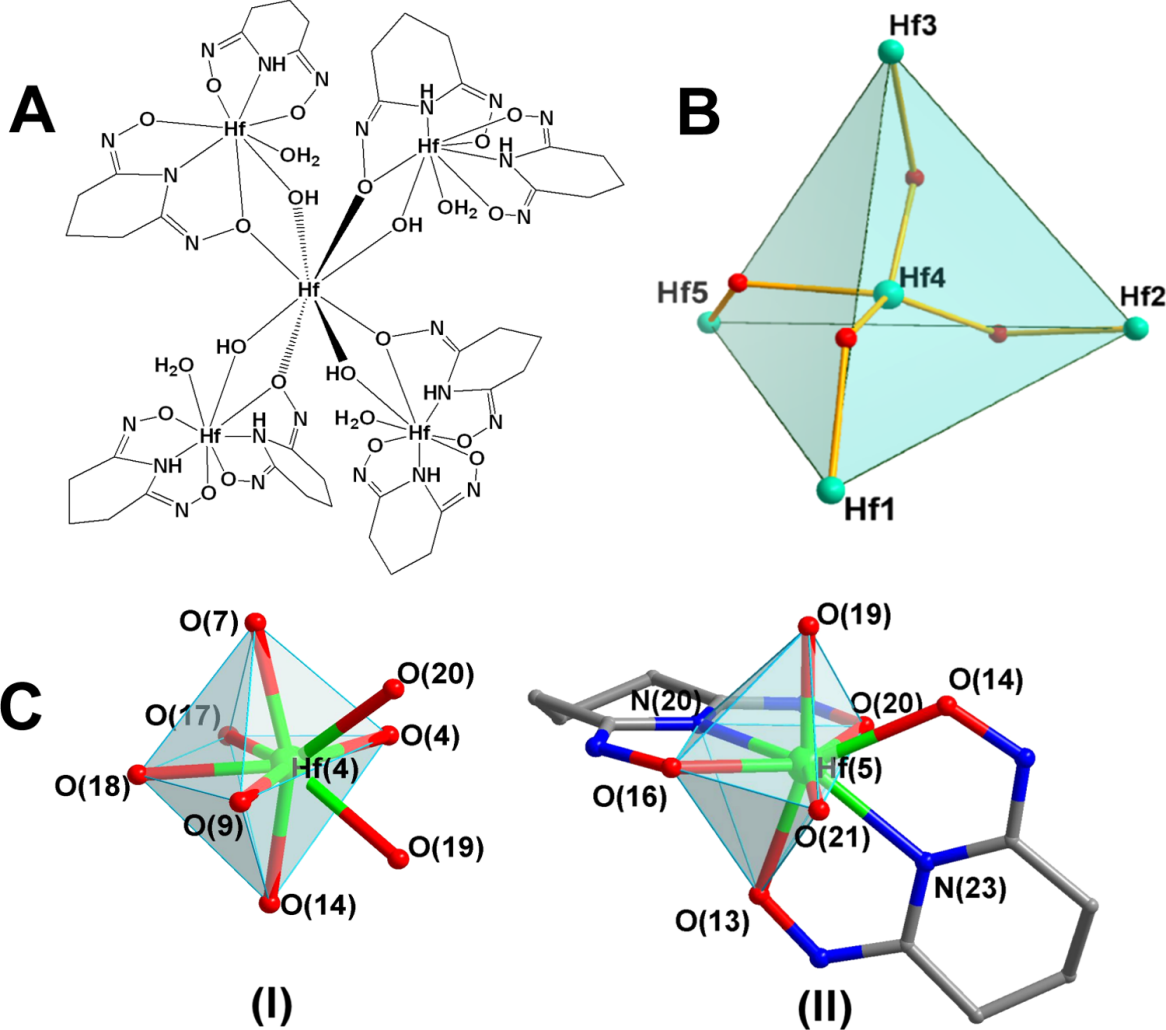
(A) Molecular drawing of the neutral pentanuclear HfOC **1**, [Hf_5_(μ-OH)_4_(μ-Hpidiox)_4_(Hpidiox)_4_]. (B) Tetrahedral arrangement of [Hf_5_^IV^(μ-OH)_4_]. (C) The bi-capped octahedral coordination environment of Hf(4) (**I**) and Hf(5) (**II**) atoms in **1**.

**Figure 2:**
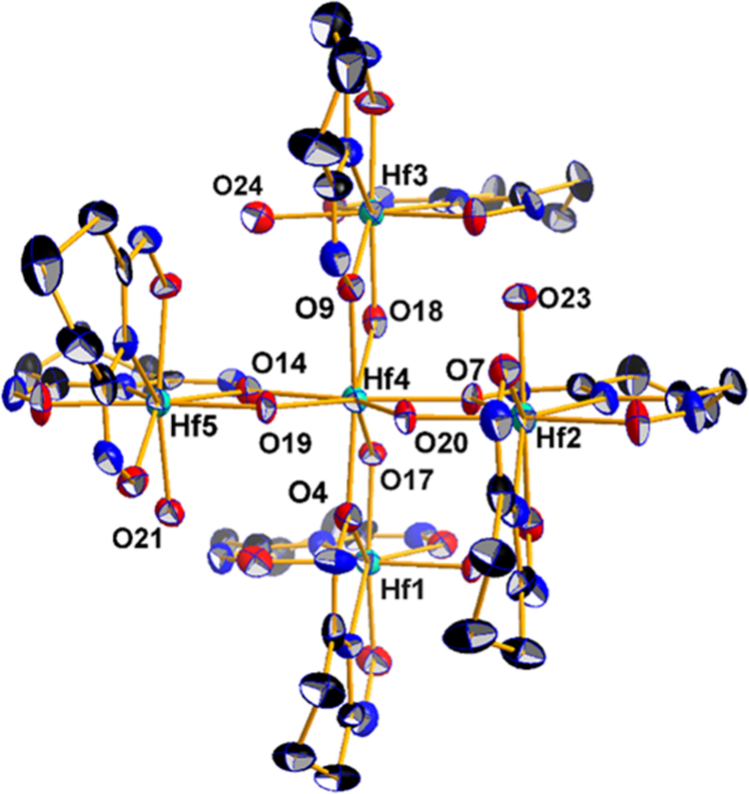
Oak Ridge thermal ellipsoid plot (ORTEP) (50% probability level) of the neutral pentanuclear HfOC **1**, with a partial labeling scheme. Hydrogen atoms and co-crystallized solvent molecules have been omitted for clarity.

The structure of HfOC **1** contains a pentanuclear hafnium(IV) core supported by four chelate Hpidiox^2–^ ligands, four chelate-bridging Hpidiox^2–^ and four μ_2_-OH^–^ groups (Figure 1A). Interestingly, the {Hf_5_^IV^} cluster is the only single pentanuclear hafnium(IV) cluster reported so far.^9^ The four outer hafnium(IV) atoms adopt a distorted tetrahedral arrangement (Figure 1B). In the center of the {Hf_4_^IV^} tetrahedron is located the fifth hafnium(IV) atom, Hf(4) (Figure 1B), bound by four μ-bridging oxime oxygen atoms and four μ_2_-OH^–^ groups in a bi-capped distorted O_8_ coordination (Figure 1A,C-I). The four outer hafnium(IV) atoms also adopt a bi-capped distorted N_2_O_6_ ligation (Figure 1C-II). Based on bond valence sum (BVS) calculations, the valences of O(17), O(18), O(19), and O(20) atoms were found to be close to 2 after considering a proton attached to each oxygen atom. BVS calculations for the terminal oxygen atoms O(21), O(22), O(23), and O(24) (Figure 2) revealed that they are doubly protonated (aqua ligands). The Hf^IV^–OH_2_ bond lengths are 2.192 ± 0.003 Å. The central hafnium(IV) atom, Hf(4), shows two sets of Hf(4)^IV^–O bonds with mean Hf(4)^IV^–O bond lengths of 2.159 ± 0.007 Å for the bridging μ-OH^–^ groups and 2.21 ± 0.01 Å for the bridging oxime μ-O^–^ atoms (Figure 2).

**Figure 3:**
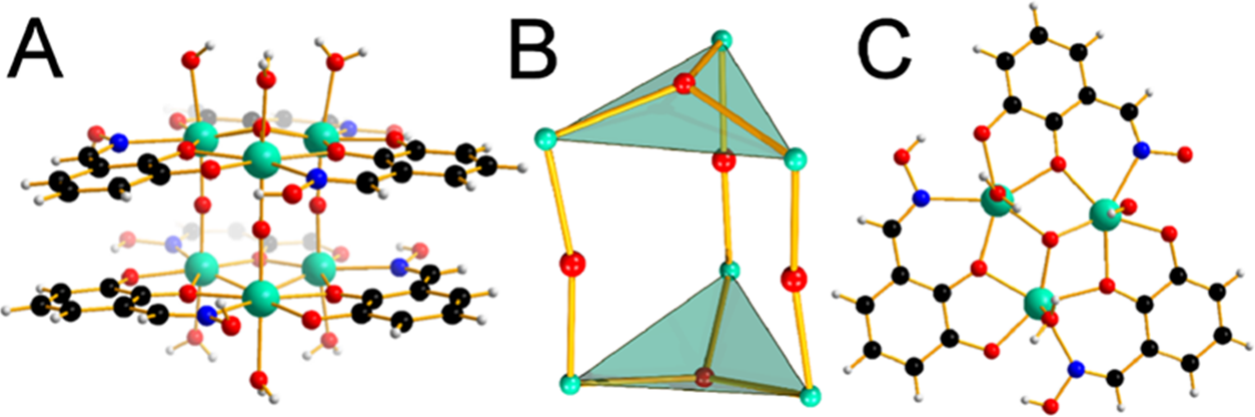
Ball-and-stick plot of cation of **3** (A). The trigonal-prismatic arrangement of the six hafnium(IV) atoms in the cluster core {Hf_6_^IV^O_5_} (B) Ball-and-stick plot of the structural unit [Hf_3_^IV^(μ_3_-O)(μ-η^1^:η^2^:η^1^-Hdihybo-*O,O*′,*N*)_3_(OH_2_)_3_] (C).

The X-ray crystallographic study of HfOC **3**, which crystallizes in a centrosymmetric space group and the unit cells contain a hexanuclear molecular structure (Figure 3A). The core structure of the hexanuclear {Hf_6_} hafnium(IV) cluster is formed from two [Hf_3_^IV^(μ_3_-O)] subunits, which are connected by three μ-O^2–^ bridges with the six hafnium(IV) atoms in a trigonal-prismatic arrangement (Figure 3B). The {Hf_3_^IV^} subunits are supported by three chelate-bridging doubly deprotonated Hdihybo^2–^ ligands (Figure 3C).

The only hexanuclear HfOCs {Hf_6_} reported thus far that incorporate six metal atoms in three different structural arrangements are shown in Figure S2; more specifically: the octahedral (Figure S2A),^73^ the star-shaped cyclic planar (Figure S2B),^74^ and the cyclic planar (Figure S2C)^75^ arrangements of the six hafnium atoms. Thus, the trigonal-prismatic arrangement of the six hafnium atoms found in **3** is unique.

The μ_3_-O^2–^ is located 0.489(4) Å above the plane defined by the three hafnium(IV) atoms Hf(1)–Hf(2)–Hf(3) (Figure 3B). The distances between the three Hf atoms bridged by the μ_3_-O^2–^ atom is 3.455 ± 0.004 Å. The distance between the two trigonal planes defined by Hf(1)–Hf(2)–Hf(3) and atoms Hf(4)–Hf(5)–Hf(6) is 3.866 (4) Å. The Hf^IV^–μ_3_-O^2–^ bond lengths are 2.053 ± 0.003 Å, while the Hf−μ-O^2–^ bond lengths are 1.929 ± 0.002 Å. The three angles in the symmetry-related Hf_3_^IV^ triangles are almost 60°, and thus, it is obvious that the Hf_3_^IV^ triangles are equilateral. The *d*(Hf^IV^–*O*_cat_^–^)_av_ and *d*[Hf^IV^–(μ-*O*_cat_)]_av_ values are 2.120(4) and 2.217(4) Å, respectively, and are very close to those reported in the literature.^76^ The doubly deprotonated catecholate moiety adopts a singly bridging chelate μ-(*O*,*O*′,*O*′) mode of coordination. The *d*(Hf^IV^–*N*_ox_)_av_ value of 2.384(6) Å is much higher than the reported mean value of 2.187(8) Å,^77^ but in the latter case, the oxime is deprotonated and acts as a μ_2_-η^1^,η^2^-*N*,*O*^–^ bridging ligand. Compound **3** is the first example of a polyoxocatecholate hafnium(IV) compound reported to date.

HfOCs **2** and **4** contain identical {Hf_6_} structural units to cluster **3**. Thus, only the structural features of compound **3** are discussed in detail (vide supra). In the case of HfOC **2**, its structure was not possible to be finalized due to severe disorder of the tetrabutyl ammonium counterions as can be seen in Figure S1. Nevertheless, the remaining part of the structure is well resolved. Additionally, the X-ray structure analysis of **4** revealed a unit cell with two {Hf_6_} clusters, where one of them was found to accommodate two methoxy terminal ligands and four aqua ligands instead of six aqua ligands (Figure S3). The structural features of **4**’s metallic core are identical to those of **3**.

### Solid-State UV–Vis Spectroscopy

**Figure 4:**
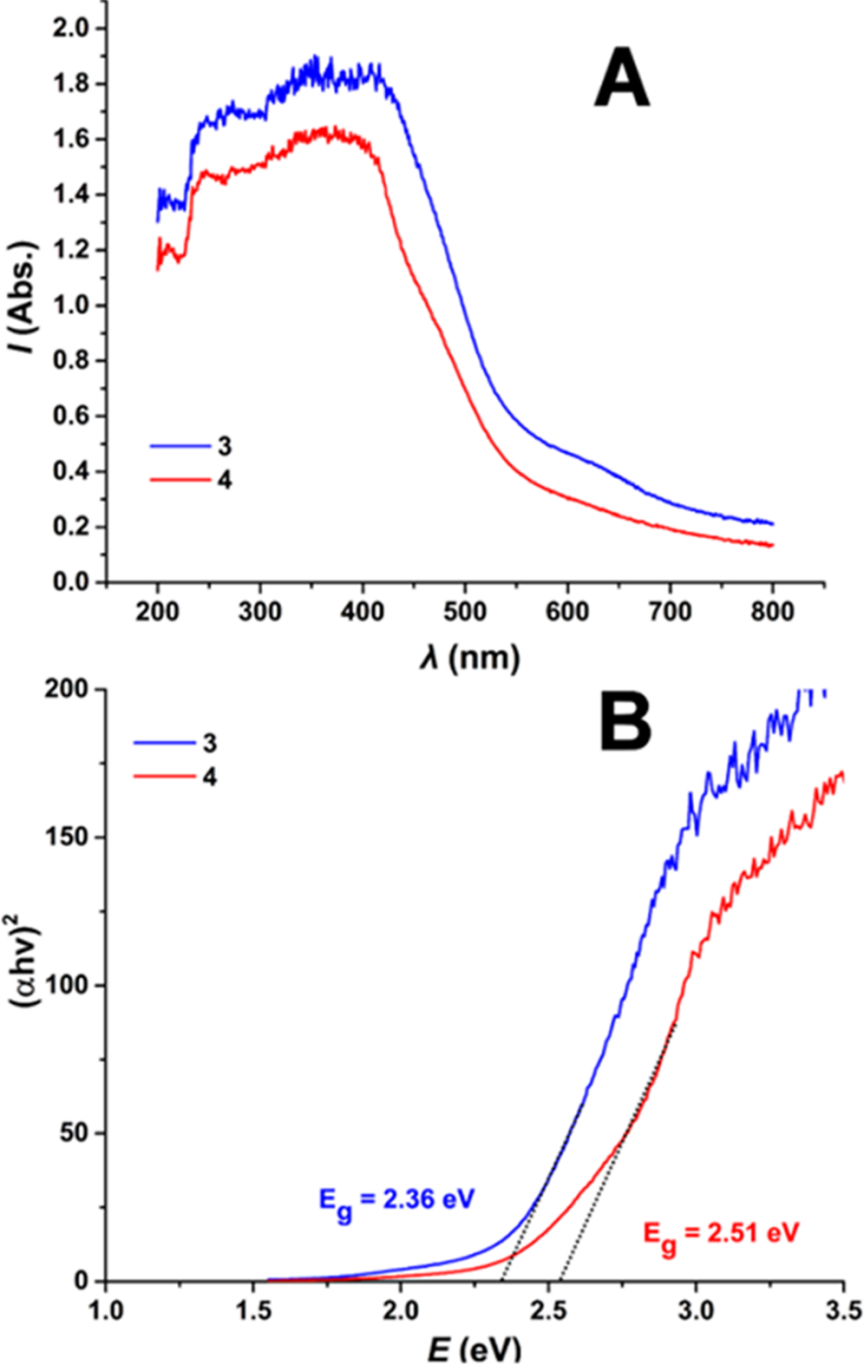
(A) UV–vis reflectance spectra of HfOCs **3** and **4**. (B) Tauc plots of HfOCs **3** and **4**.

Figure 4 shows the solid-state UV–vis spectra of HfOCs **3** and **4**, while the solution (MeOH) UV–vis spectra of **1** and **3** are shown in Figures S4–S6. In solution, **1** and **3** absorb in the UV region up to 360 and 500 nm, respectively. In the solid state the absorption of **3** and **4** shifts to lower energy up to 800 nm due to the intermolecular interactions. The band gaps for compounds **3** and **4** were found to be 2.36 and 2.51 eV, respectively, and were calculated from the solid-state spectra employing the Kubelka–Munk method.^78^ Interestingly, HfOCs **3** and **4** revealed substantially low band gap values rendering the Hf-based molecular species highly promising candidates for semiconducting photocatalytic applications. This finding demonstrates the constructive co-operative effect of the catechol and oxime moieties not only in stabilizing unique structural features but also in modifying the clusters’ electronic structure by substantially reducing the band gap value in comparison to Hf^IV^O_2_. This observation paves the way for the discovery of new HfOCs by exploiting the stabilizing effect of organic ligands that incorporate appropriate coordinating moieties.

### NMR Spectroscopy

**Figure 5:**
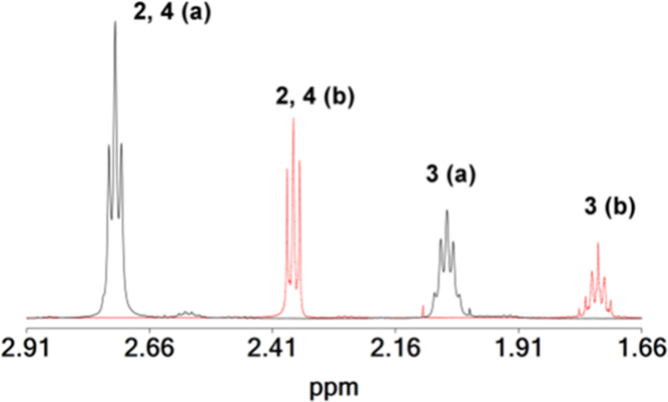
^1^H NMR spectra in solution (CD_3_OD) of the pentanuclear cluster **1** (in black color) and of the H_3_pidiox ligand (in red color).

**Scheme 3:**
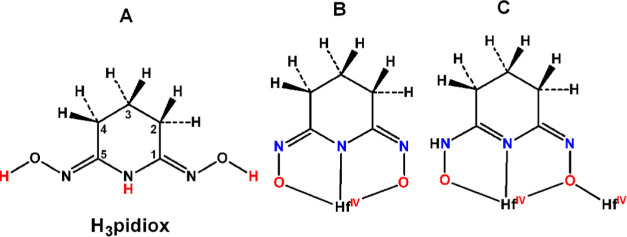
Numbering of the Carbon Atoms of the Ligand H_3_pidiox (A) and the Two Modes of Ligation (B, C) of Hpidiox^2–^ in **1**

The ^1^H and ^13^C NMR chemical shifts of the ligand H_3_pidiox and the pentanuclear hafnium(IV) cluster **1** in solution (CD_3_OD) are collected in Table 1. The ^1^H NMR spectrum of the ligand H_3_pidiox (Figure 5) shows one quintet and one triplet at 1.71 and 2.37 ppm assigned to the two protons attached to C(3) and the four protons attached to {C(2)_,_ C(4)}, respectively (Scheme 3A), while its ^13^C NMR spectrum gave three peaks at 19.32, 26.20, and 148.57 ppm assigned to C(3), {C(2), C(4)}, and {C(1), C(5)}, respectively.

**Table 1:** ^1^H and ^13^C Chemical Shifts (ppm) of the Pentanuclear Cluster **1** and the Ligand H_3_pidiox

	**1**		H_3_pidiox	
	^13^C	^1^H^a^	^13^C	^1^H^a^
C(2,4)	20.30	2.7271	26.20	2.3670
C(3)	17.97	2.0569	19.31	1.7111
C(1,5)	156.42		148.57	

a The chemical shifts of the protons are measured at the center of the observed multiplets.

**Scheme 4:**
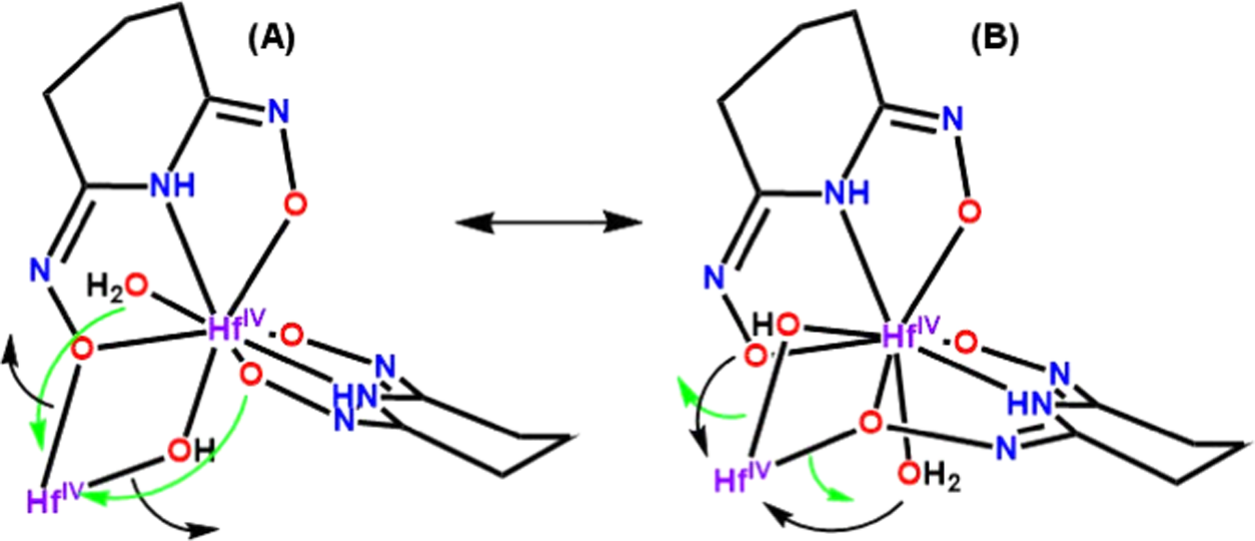
Dynamic Equilibrium between the Chelate/Bridging and Chelate Ligation Modes of the Ligand Hpidiox^2–^ in **A** from Chelate to Chelate/Bridging in **B** and Vice Versa

The X-ray diffraction analysis of **1** (Figures 1A and 2) revealed the existence of chelate (Scheme 3B) and chelate/bridging (Scheme 3C) modes of ligation of the ligand Hpidiox^2–^. However, the ^1^H and ^13^C NMR spectra of **1** exhibit only one set of peaks. This might be attributed to a fast exchange between the ligands with a different coordination mode. A dynamic structural change between the chelate and chelate/bridging modes can occur intramolecularly by a simple breaking of the Hf(1)–O_oxime/bridging_ bond (Scheme 4A) and the formation of an Hf(1)–O_oxime/terminal_ bond (Scheme 4B) and vice versa.

The ^13^C NMR peaks of the carbon atoms 2, 4, and 3 in cluster **1** [identified in two-dimensional (2D) {^1^H,^13^C} grHSQC spectrum, Figure S7] shift to a higher field, 17.97 C(3), 20.30 C(2, 4), and of C(1, 5) [156.42, identified in the 2D {^1^H,^13^C} grHMBC spectrum (Figure S8)] to a lower field compared with the chemical shifts of the free ligand (Table 1).

**Scheme 5:**
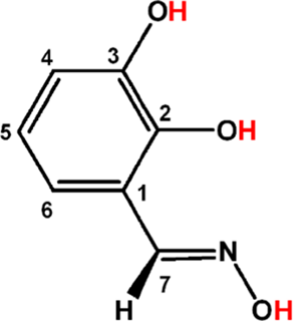
Numbering of the Carbon Atoms of the H_3_dihybo Ligand

The ^1^H and ^13^C NMR peaks of the free ligand are also shifted in the hexanuclear HfOCs **3** or **4** due to the ligation of Hdihybo^2–^ to the Hf^IV^ metal centers. The coordination of the metal ion to the oxime nitrogen atom induces deshielding of C(7) (Scheme 5) for **3** (0.3 ppm, Table 2).

**Table 2:** ^13^C [^1^H] NMR Chemical Shifts for H_3_dihybo and **3** and the Shielding/Deshielding Effect (Δδ, ppm) upon Complexation

	H_3_dihybo	**3**/δ^13^C^a^ (Δδ^b^) [δ^1^H (Δδ^b^)]
C(1)	117.6	
C(2)	146.5	
C(3)	146.4	
C(4)	120.4 [6.80]	120.5 (+0.1) [6.804 (+0.004)]
C(5)	118.8 [6.72]	122.0 (+3.2) [6.674 (−0.046)]
C(6)	121.7 [6.76]	124.5 (+2.8) [6.231 (−0.529)]
C(7)	152.3 [8.19]	152.6 (+0.3) [7.781 (−0.410)]

a The ^13^C NMR chemical shifts of **3** were found from the 2D {^1^H,^13^C} grHSQC spectrum.

b δ_M–L_ – δ_L_.

**Figure 6:**
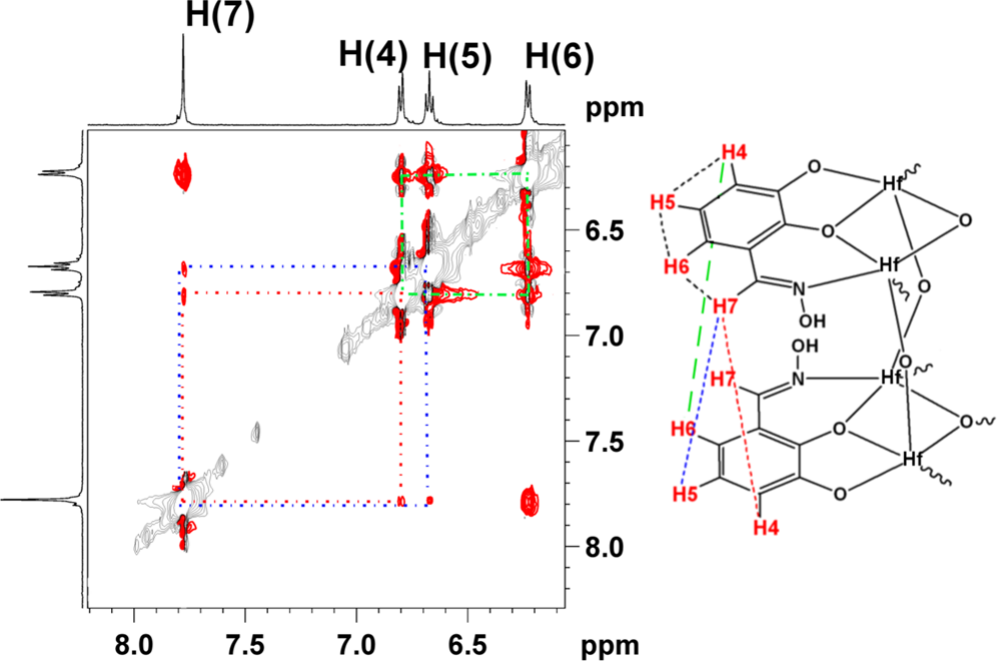
Two-dimensional (2D) {^1^H} NOESY spectrum of HfOC **3** and the assignments of the NOESY interactions.

The ligand’s H_3_dihybo neighboring protons of **3** in solution (CD_3_OD) [H(7)–H(6), H(6)–H(5), H(5)–H(4)] show strong NOESY interaction in 2D {^1^H} nuclear Overhauser effect spectroscopy (NOESY)–exchange spectroscopy (EXSY) spectra. Weaker NOESY off-diagonal peaks (Figure 6) are observed between the protons H(7)–H(4), H(7)–H(5), and H(4)–H(6) assigned to intra-ligand proton interactions between the protons of the ligands belonging to the two [M_3_(Hdihybo)_3_] planes as depicted for HfOC **3** in Figure 6. The distances between protons 4–7, based on the intensity of the NOESY cross peaks, are H(7)–H(4) < H(4)–H(6) < H(7)–H(5), in agreement with the distances found in the crystal structure of HfOC **3**. Apparently, HfOCs **3** and **4** retain their structural integrity in solution. The ^1^H and ^13^C NMR spectra of HfOCs **3** and **4** in D_2_O and CD_3_OD are similar indicating that HfOCs **3** and **4** are hydrolytically stable.

### Electrospray Ionization Mass Spectrometry (ESI-MS)

The characterization of the pentanuclear {Hf_5_} cluster **1** in solution was carried out using high-resolution ESI-MS to determine the compound’s stability in solution.^79^ The ESI-MS studies were performed in methanol in negative ionization mode. The presence of higher-intensity isotopic distribution envelopes is due to the existence of the pentanuclear moiety, resulting from the combination of protons, counterions, and solvent molecules. Occasionally, the in situ induction of partial fragmentation during the course of ion transfer in addition to alteration of the metal’s oxidation state is quite common and has been reported frequently in the literature.^80,81^ A series of doubly charged distribution envelopes that can be assigned to the intact {Hf_5_} cluster (Figure S9) can be observed in the higher region of *m*/*z* values. In this case, a group of distribution envelopes are clustered within the range of ca. 1100–1300 *m*/*z*. More specifically, the central isotopic distribution envelope at 1191.95 *m*/*z* can be assigned to the {Hf_5_^IV^(C_5_H_6_N_3_O_2_)_8_(OH)_4_K_6_(OH_2_)_4_}^2–^ anion which is flanked by isotopic envelopes of the same moiety associated with a different number of solvent molecules with the general formula of {Hf_5_(C_5_H_6_N_3_O_2_)_8_(OH)_4_K_6_(OH_2_)*_x_*}^2–^, where *x* = 0–12 (Table 3). A representative example of this group of species is the expanded distribution envelope shown in Figure S10 along with its simulated pattern, which corresponds to the intact {Hf_5_} cluster. In the range of ca. 1500–1600 *m*/*z* values, another group of envelopes has been identified and assigned as a singly charged tetrameric cluster, which is due to the partial fragmentation of the {Hf_5_} moiety which takes place during the ionization and ion transfer process^80,81^ with general formula {Hf_4_^IV^L_4_K*_y_*(OH_2_)*_z_*(OCH_3_)_7_}^−^, where *y* = 0, 1 and *z* = 0, 2, 3, or 4 (Table 3).

**Table 3:** Representation of the Experimentally Identified and Simulated *m*/*z* Values of the Distribution Envelopes of {Hf_5_} Cluster **1**

exp.	theor.	charge	formula
1528.96	1529.04	–1	{Hf_4_^IV^(C_5_N_3_O_2_H_6_)_2_(C_5_N_3_O_2_H_5_)_2_K(OCH_3_)_7_}^−^
1565.93	1566.07	–1	{Hf_4_^IV^(C_5_N_3_O_2_H_6_)_3_(C_5_N_3_O_2_H_5_)K(OH_2_)_2_(OCH_3_)_7_}^−^
1584.92	1585.08	–1	{Hf_4_^IV^(C_5_N_3_O_2_H_6_)_4_K(OH_2_)_3_(OCH_3_)_7_}^−^
1602.90	1603.09	–1	{Hf_4_^IV^(C_5_N_3_O_2_H_6_)_4_K(OH_2_)_4_(OCH_3_)_7_}^−^
1155.96	1155.92	–2	{Hf_5_^IV^(C_5_N_3_O_2_H_6_)_4_(C_5_N_3_O_2_H_5_)_4_(OH)_4_K_6_}^2–^
1173.95	1173.93	–2	{Hf_5_^IV^(C_5_N_3_O_2_H_6_)_4_(C_5_N_3_O_2_H_5_)_4_(OH)_4_K_6_(OH_2_)_2_}^2–^
1191.94	1191.94	–2	{Hf_5_^IV^(C_5_N_3_O_2_H_6_)_4_(C_5_N_3_O_2_H_5_)_4_(OH)_4_K_6_(OH_2_)_4_}^2–^
1211.92	1211.97	–2	{Hf_5_^IV^(C_5_N_3_O_2_H_6_)_8_(OH)_4_K_6_(OH_2_)_6_}^2–^
1229.90	1229.98	–2	{Hf_5_^IV^(C_5_N_3_O_2_H_6_)_4_(OH)_4_K_6_(OH_2_)_8_}^2–^
1248.38	1248.50	–2	{Hf_4_^IV^Hf^III^(C_5_N_3_O_2_H_6_)_8_H(OH)_4_K_6_(OH_2_)_10_}^2–^
1266.86	1267.00	–2	{Hf_3_^IV^Hf_2_^III^(C_5_N_3_O_2_H_6_)_8_H_2_(OH)_4_K_6_(OH_2_)_12_}^2–^

**Figure 7:**
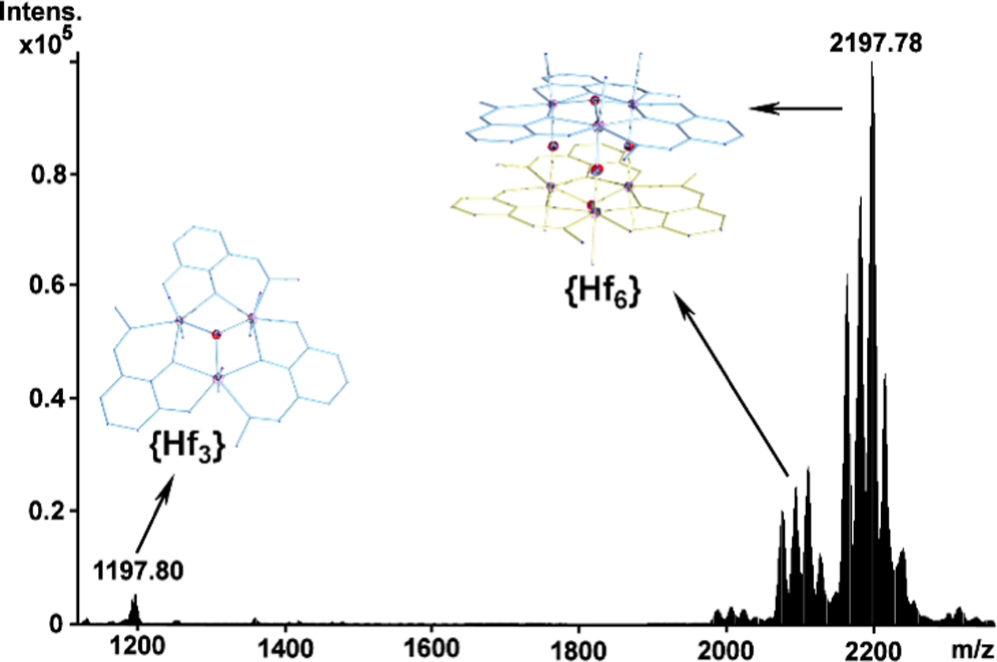
Negative ion mass spectrum of **3** exhibiting two characteristic sets of isotopic distribution envelopes centered at ca. 1179.80, 1183.80, and 1193.85 *m*/*z* with the formulae {Hf_3_O(C_7_H_5_NO_3_)_2_(C_7_H_4_NO_3_)(OH_2_)_7_Cl(HOMe)}^−^, {Hf_3_O(C_7_H_5_NO_3_)_2_(C_7_H_4_NO_3_)(OH_2_)_8_Cl}^−^, and {Hf_3_O(C_7_H_5_NO_3_)_2_(C_7_H_4_NO_3_)(OH_2_)_5_Cl(HOMe)_2_}^−^, respectively, and in the range of ca. 2000–2300 *m*/*z* with the general formula {Hf_6_^IV/III^O_5_L_6_(OH_2_)*_z_*(OCH_3_)*_y_*Cl*_z_*H*_w_*}^−^, where *x* = 1–8, *y* = 0 or 1, *z* = 0 or 1, and *w* = 1–5.

In a similar manner, the {Hf_6_} cluster preserves its structural features in solution, as identified by a series of singly charged distribution envelopes (Figure 7). The assignment of the observed isotopic distribution envelopes reveals the main structural motif {Hf_3_^IV^Hf_3_^III^O_5_(C_7_H_5_NO_3_)_3_(C_7_H_4_NO_3_)_3_}(solv)*_x_* associated with different amalgamations of solvent molecules (H_2_O or CH_3_OH) coordinated or associated with the ionized {Hf_6_} cluster, see Table 4 for a detailed assignment. In a similar fashion, the observation of changes in the metal’s oxidation state is quite common as discussed above.

**Table 4:** Representation of the Experimentally Identified and Simulated *m*/*z* Values of the Distribution Envelopes of {Hf_6_} Cluster **3**

exp.	theor.	charge	formula
2075.70	2075.81	–1	{Hf_3_^IV^Hf_3_^III^O_5_(C_7_H_5_NO_3_)_3_(C_7_H_4_NO_3_)_3_(OH_2_)H_3_}^−^
2094.69	2094.83	–1	{Hf_2_^IV^Hf_4_^III^O_5_(C_7_H_5_NO_3_)_3_(C_7_H_4_NO_3_)_3_(OH_2_)_2_H_4_}^−^
2110.69	2110.83	–1	{Hf_4_^IV^Hf_2_^III^O_5_(C_7_H_5_NO_3_)_3_(C_7_H_4_NO_3_)_3_(OH_2_)_3_H_2_}^−^
2127.73	2127.83	–1	{Hf_5_^IV^Hf^III^O_5_(C_7_H_5_NO_3_)_3_(C_7_H_4_NO_3_)_3_(OH_2_)_4_H}^−^
2163.79	2163.89	–1	{Hf^IV^Hf_5_^III^O_5_(C_7_H_5_NO_3_)_3_(C_7_H_4_NO_3_)_3_(OH_2_)_4_(HOMe)H_5_}^−^
2181.77	2181.89	–1	{Hf^IV^Hf_5_^III^O_5_(C_7_H_5_NO_3_)_3_(C_7_H_4_NO_3_)_3_(OH_2_)_5_(HOMe)H_5_}^−^
2197.78	2197.89	–1	{Hf_3_^IV^Hf_3_^III^O_5_(C_7_H_5_NO_3_)_3_(C_7_H_4_NO_3_)_3_(OH_2_)_6_(HOMe)H_3_}^−^
2214.78	2214.90	–1	{Hf_4_^IV^Hf_2_^III^O_5_(C_7_H_5_NO_3_)_3_(C_7_H_4_NO_3_)_3_(OH_2_)_7_(HOMe)H_2_}^−^
2238.74	2238.87	–1	{Hf_2_^IV^Hf_4_^III^O_5_(C_7_H_5_NO_3_)_3_(C_7_H_4_NO_3_)_3_(OH_2_)_8_ClH_5_}^−^

Interestingly, the lower *m*/*z* region (ca. 1000–1200 *m*/*z*) revealed the presence of the fundamental trimeric building block generated during the ionization process. The singly charged distribution envelopes centered at 1179.80, 1183.80, and 1193.85 *m*/*z* values demonstrate the presence of the Hf-oxo-centered triangles that can provide crucial information about the formation of these clusters. The identification of the trimeric building blocks indicates the formation of the trimeric clusters prior to their subsequent utilization as building blocks for the construction of the hexanuclear species that can be isolated as single crystals. Similar behavior has been observed in the solution studies conducted for the {Ti_6_} and {Zr_6_} species reported previously by our group.^40,43^ More specifically, we observed the {Hf_3_O(C_7_H_5_NO_3_)_2_(C_7_H_4_NO_3_)(OH_2_)_7_Cl(HOMe)}^−^ (1179.80), {Hf_3_O(C_7_H_5_NO_3_)_2_ (C_7_H_4_NO_3_)(OH_2_)_8_Cl}^−^ (1183.80), and {Hf_3_O(C_7_H_5_NO_3_)_2_(C_7_H_4_NO_3_)(OH_2_)_5_Cl (OHMe)_2_}^−^ (1193.85) Hf-based oxo-centered molecular triangles, as shown in Figure S11.

### Metalloaromaticity of the Cyclic Trinuclear {Hf_3_} Metallic Ring Cores of HfOC **3** {Hf_6_}

**Figure 8:**
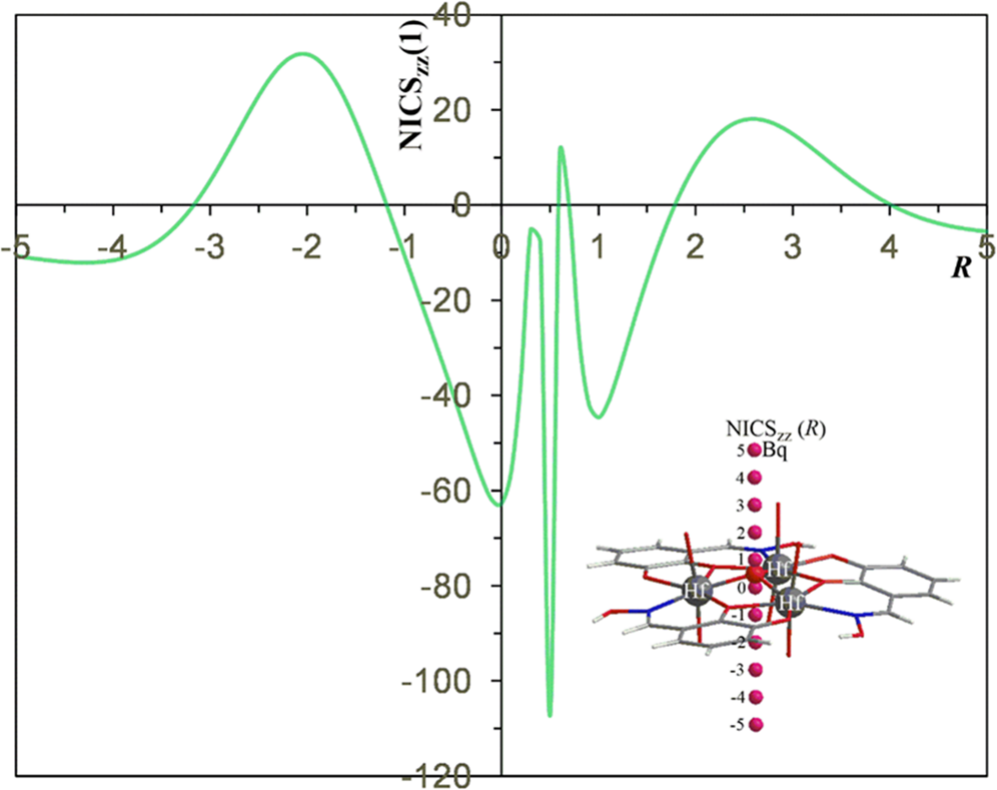
NICS*_zz_*(*R*) (*R* in Å) scan profile of *cyclo*-{Hf_3_} trinuclear core calculated at the GIAO/PBE0/Def2-TZVP(Hf)U6-31G(d,p)(E) level.

Next, we set out to study whether or not the cyclic trinuclear {Hf_3_} metallic ring cores exhibit metalloaromaticity^50^ and to probe the aromaticity/antiaromaticity of the rings, we employed the magnetic criterion, i.e., the nucleus-independent chemical shifts (NICS) by computing the NICS*_zz_* scan curves.^82−84^ Accordingly, we calculated the NICS*_zz_* scan curve of the *cyclo*-Hf_3_ trinuclear metallic core with the PBE0 functional that has been found to perform well in modeling molecular properties of heavy metals.^85^ The NICS*_zz_* curve is given in Figure 8, while the inset picture depicts the positions of the Bq ghost atoms. Sigma aromaticity arises from σ MOs delocalized in the plane of the ring, while π, δ, and ϕ arise from π, δ, and ϕ MOs delocalized over the ring plane, thus, σ type aromaticity is expressed by NICS*_zz_* in the center of the plane [NICS*_zz_*(0)], while π, δ, and ϕ type aromaticities are expressed by NICS*_zz_* 1 Å above the center of the plane [NICS*_zz_*(1)].

Note that the NICS*_zz_* scan curve given in Figure 8 was calculated for the structure obtained from the X-ray structural analysis of this system. The NICS*_zz_*(1) (Bq located 1 Å above the {Hf_3_} core) is equal to −44.6 ppm indicative of aromaticity. The magnetic aromaticity of the {Hf_3_} core is comparable to that of benzene for which the NICS*_zz_*(1) is calculated to be equal to −29.9 ppm at the GIAO/PBE0/6-31G(d,p) level. Also, the {Hf_3_} core is more aromatic compared to a similar system bearing a {Zr_3_} core and exhibiting NICS*_zz_*(1) equal to −37.3 ppm.^40^ However, the presence of the O ligand capping the {Hf_3_} ring contributes to the NICS*_zz_*(1) values and so the aromaticity of the ring should be smaller. In this context, we used the NICS*_zz_*(0) values to quantify the aromaticity of the three-member ring {Hf_3_}. Accordingly, NICS*_zz_*(0) is equal to −62.7 ppm suggesting strong σ-type metalloaromaticity of the {Hf_3_} ring core. Interestingly, the {Hf_3_} ring core exhibits a stronger metalloaromaticity than the respective {Zr_3_} ring core with a NICS*_zz_*(0) value equal to −40.1 ppm at the same level of theory.^36^ The smaller size of the {Hf_3_} ring relative to {Zr_3_} ring accounts well for the observed higher metalloaromaticity of the former (the ring radius of the {Hf_3_} ring is 1.996 Å as compared to the ring radius of 2.002 Å in the {Zr_3_}). The perusal of Figure 8 reveals that the NICS*_zz_* curve indicates the existence of multiple and alternating aromaticity/antiaromaticity zones. Away from the *cyclo*-{Hf_3_} metallic cores, at distances of 2–3 Å from the metallic ring centers, there are two antiaromatic zones with NICS*_zz_*(*R*) peak values around 32 ppm (at 2 Å below the ring plane) and around 18.1 ppm (at 2.6 Å above the ring plane). In contrast, at 0.5 near the O capping ligand (which is located 0.488 Å above the ring plane) the NICS*_zz_*(1) is extremely high with a value equal to −107.3 ppm.

### Photophysical Properties of HfOCs **1** and **3**

**Figure 9:**
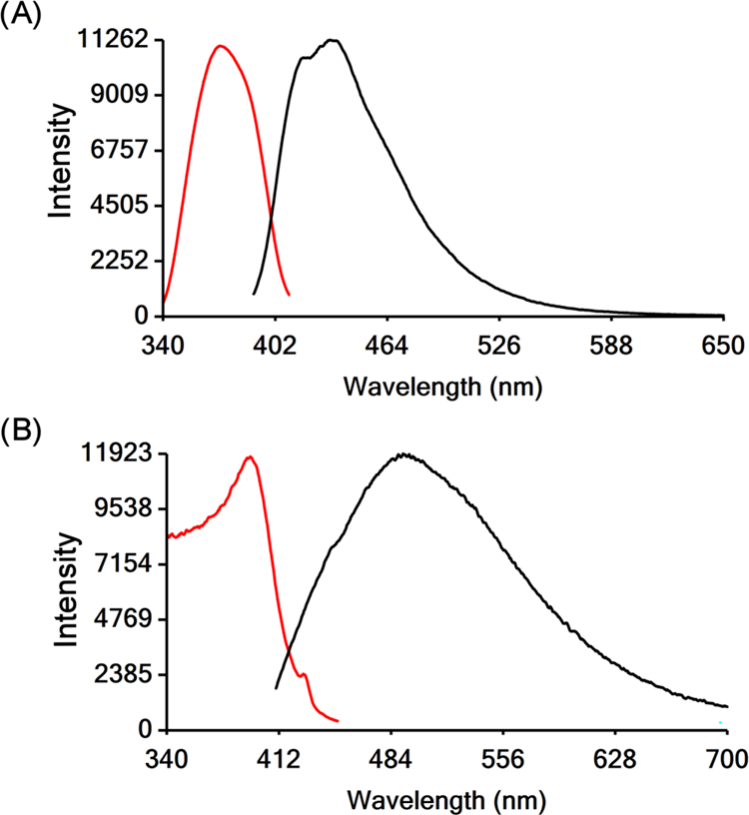
Excitation (red line) and emission (black line) spectra of (A) **1** and (B) **3** in solution (MeOH, 1.00 mM).

Taking into consideration the interesting electronic structure and decreased band gap values observed above, we embarked on exploring the luminescence properties of the H_3_dihybo and HfOCs **1** and **3** that are shown below in Table 5. The imide–dioxime organic molecule H_3_pidiox (Scheme 1) does not show any significant light emission. However, the pentanuclear HfOC **1** emits light at 455 nm upon excitation at 362 nm (Figure 9A). The excitation spectrum of HfOC **1** exhibits two excitation peaks at 362 and 400 nm. Cluster **1** has a low absorption of up to 500 nm (Figure S6). The intensity of the excitation peaks might be due to the energy transfer from the higher energy transitions of 1.^86^

**Table 5:** Luminescence Data of H_3_dihybo and the HfOCs **1** and **3** in Solution (MeOH, 1.00 mM)

compound	excitation (nm)	emission (nm)	quantum yield
H_3_dihybo	362	417	42
H_3_dihybo + 4 equiv of Et_3_N	305	420	8
**1**	362	455	87
**3**	405	506	35

The catecholate–oxime organic molecule H_3_dihybo (Scheme 1), emits light at 417 nm (Table 5) upon excitation at 362 nm.

Upon complexation of the H_3_dihybo ligand to the Hf(IV) centers in the hexanuclear HfOC **3**, both the excitation and emission maximum wavelengths are shifted to lower energy, by 60 and 100 nm, respectively (Figure 9B and Table 5). HfOC **3** emits light in the cyan-green region (505 nm). The theoretical investigation of the luminescence properties of **1** and **3** (vide infra) predicts that the emitted light of these HfOCs is due to ligand–ligand electron transitions. However, ligation of the ligands to the Hf^IV^ centers results in a significant change in intensity and the wavelength of the emitted light. Considering that both ligands are strong chelators for hard metals, these ligands can be used for the extraction of Hf^IV^ by chelation and the above luminescence properties can be employed for sensing Hf^IV^ ions in solutions.

**Figure 10:**
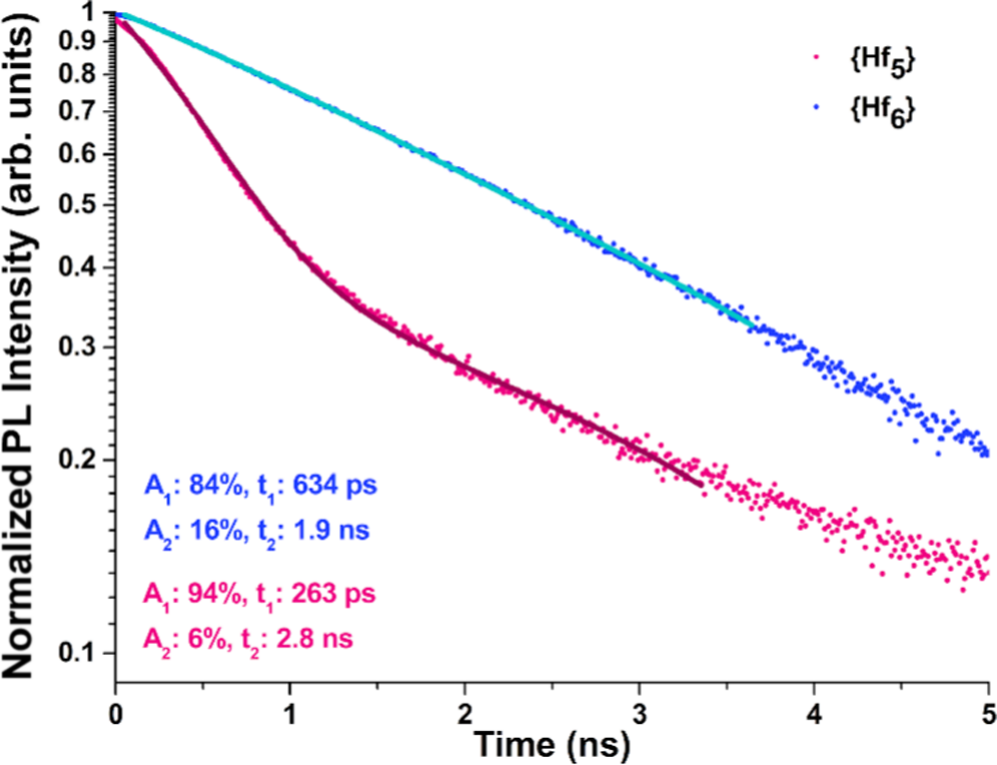
Photoluminescence decays of {Hf_6_} **3** (upper, blue) and {Hf_5_} **1** (lower, pink), shown with bi-exponential fits (solid lines) with parameters detailed in the inset and described in the main text.

In an effort to further explore the emission behavior of clusters **1** and **3**, we investigated the decay time of the excited state. In the case of compound **1** (Figure 10), the detection wavelength selected using the monochromator was 455 nm and the acquisition time was approximately 12 h to ensure a good signal-to-noise ratio as shown. The lifetime exhibits a bi-exponential decay, where the fast component dominates significantly (∼95%) at around 260 ps. The slow component makes up only 5% and is significantly slower, with a lifetime of just under 3 ns.

In the case of compound **3** (Figure 10), the detection wavelength was selected instead to be 520 nm and the acquisition time was as little as 3 h as it was slightly more emissive than the first sample. This ensured a similar signal-to-noise ratio to compound **1**. The lifetime of the hexanuclear cluster **3** also exhibits a bi-exponential decay, where the fast component also dominates (∼85%) but instead with a much slower lifetime of approximately 630 ps. Its slow component makes up only ∼15% and is instead a whole nanosecond faster than that of the previous samples’ slow decay component, with a lifetime of just under 2 ns.

### Theoretical Study of the Optical Properties of HfOCs **1** {Hf_5_} and **3** {Hf_6_}

To tap into the optical properties of both complexes full geometry optimization of their single crystal coordinates was performed. The electronic structure of the ground states is typical of high-valent oxo complexes. The highest occupied molecular orbital–lowest unoccupied molecular orbital (HOMO–LUMO) gaps in {Hf_5_} **1** and {Hf_6_} **3** clusters are thus 2.68 and 2.29 eV (see Figures S12 and S13), respectively, showing a large separation between the oxo and metal “bands”. The value of 2.29 eV is very close to that of the experimental value of 2.36 eV from the UV–vis reflectance spectrum of the {Hf_6_} **3** (vide supra).

**Figure 11:**
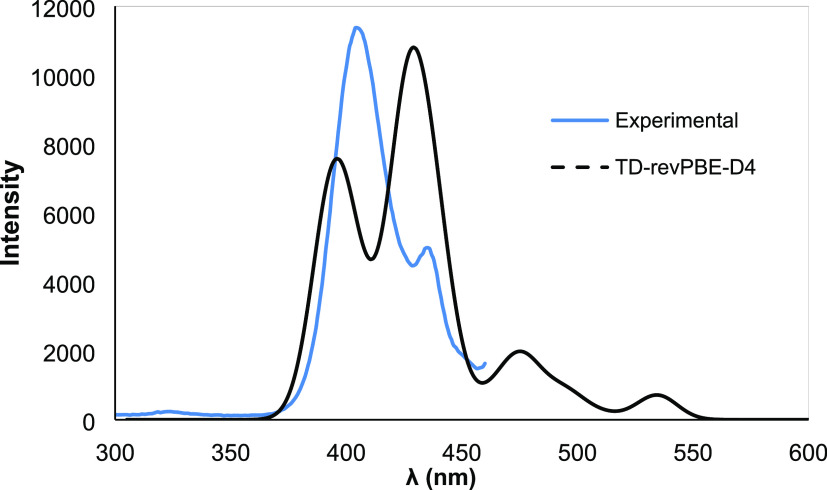
Excitation spectrum of HfOC **3** {Hf_6_} contrasted with the calculated one.

The absorption spectrum of both systems was calculated at the time-dependent DFT (TD-revPBE-D4/TZP) level. It may be seen that for {Hf_6_} **3** (Figure 11) the absorption wavelengths have excellent agreement with the experimental values although the absorption intensities are reversed. This may be an indication that in solution there may be a strong interaction with the solvent possibly through hydrogen bonds which cannot be captured by the implicit solvation scheme, and this leads consequently to symmetry breaking.

**Figure 12:**
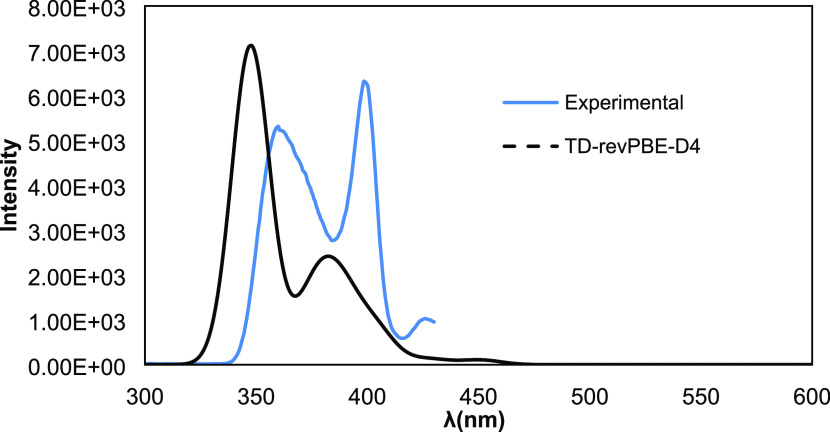
Excitation spectrum of HfOC **1** {Hf_5_} contrasted with the calculated one.

For {Hf_5_} **1** (Figure 12) the agreement is not so good, and the bands are uniformly UV-shifted by 20–30 nm and their relative intensities are also reversed. Again, this may indicate relaxation effects and strong interaction with the solvent, inaccuracies in the level of theory, or the presence of protonation equilibria on the part of the hydroxo groups.

As the excitation source of the experiment is 405 nm this coincides with the first major band in {Hf_6_} and the second one in {Hf_5_}. As such the corresponding excited states that originate from those regions of the spectrum were optimized also by the TD-revPBE-D4 methodology.

In the case of {Hf_6_}, the geometries of both the ground and excited states were optimized in global *C*_2_ symmetry meaning that these will have A and B irreducible representations. Its absorption maximum (405 nm) corresponds to the 24th excited states in either symmetry representation, herein labeled as 24(^1^A) and 24(^1^B) and these are strictly degenerate in the ground state geometry. These electronic transitions are ascribed to the molecular orbitals that are mostly centered on the ligand. For this case, it was decided to pursue the relaxation of the 24(^1^A) state.

In the case of {Hf_5_}, the 20(^1^A) and 20(^1^B) states are not strictly degenerate but are still symmetry-related counterparts and are the origin of the first band maximum in the absorption region of 400 nm. The 20(^1^A) root was therefore optimized.

In both {Hf_5_} and {Hf_6_} the targeted excited states have a ligand-to-ligand charge transfer (LLCT) character. The essential parameters are summarized in Table 6.

**Table 6:** Calculated Optical Parameters for HfOCs **1** {Hf_5_} and **3** {Hf_6_}

	{Hf_6_}	{Hf_5_}
state index	24(^1^A)	20(^1^A)
oscillator strength *f*_OSC_	0.0144	0.0435
Franck–Condon (vertical) λ (nm)	404	388
emissive λ (nm)	515 (exp. 506)	443 (exp. 455)
adiabatic Δ*E* = *E*_ES_ – *E*_GS_ (eV)	2.801	2.799
radiative lifetime τ (s)	6.21 × 10^–5^	5.36 × 10^–6^
composition	42% NTO1 → NTO2	63% NTO1 → NTO2
	25% NTO3 → NTO4	22% NTO3 → NTO4

**Figure 13:**
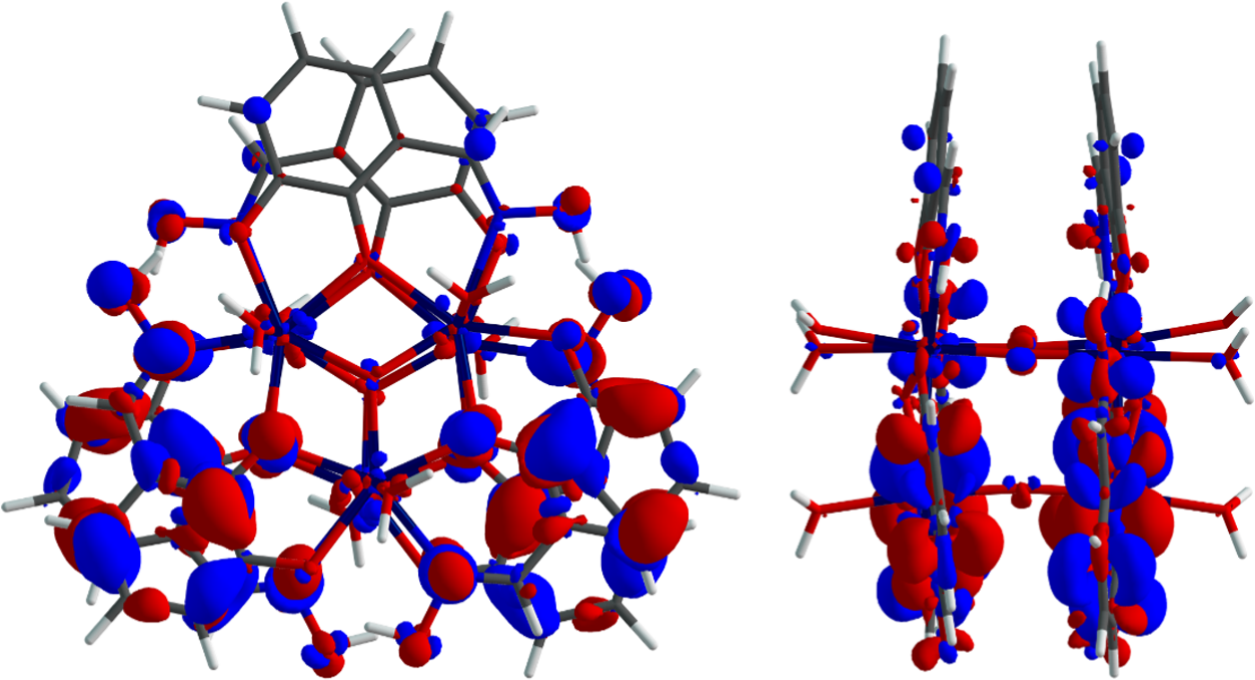
Top and side views of the calculated transition density of the emissive state of {Hf_6_}. Blue regions represent electron depletion and red regions electron gain.

The transition density of {Hf_6_} reflects the transition to and from the ligands’ π orbital manifold (Figure 13). The associated natural transition orbitals for state 24(^1^A) reflect a multi-configurational character with the majority of the transition being NTO1 → NTO2 (Table 6) while the second largest contribution stems from the NTO3 → NTO4 transition (Figure S14).

**Figure 14:**
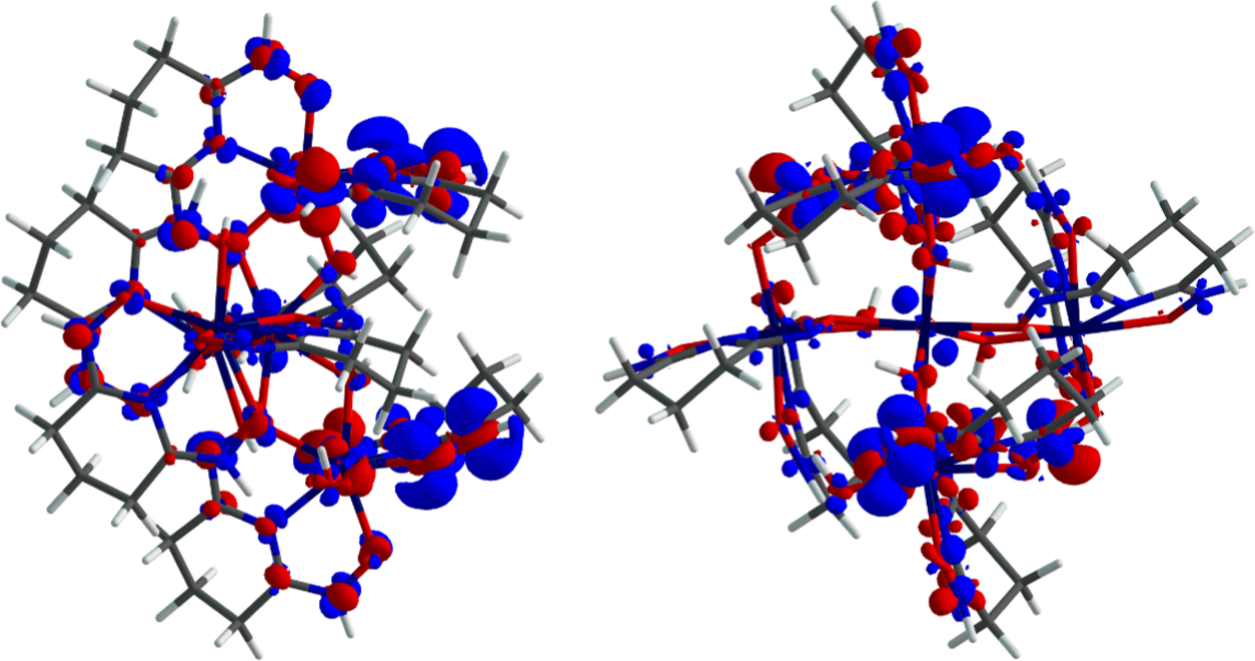
Two profile views of the calculated transition density of the emissive state of {Hf_5_}. Blue regions represent electron depletion and red regions electron gain.

In the case of {Hf_5_}, the transitions also exhibit an LLCT character (Figure 14) yet with a slightly more subtle metal 5d orbital involvement. The overwhelming contributions of the 20(^1^A) state stem from NTO1 → NTO2 (Table 6) with a smaller contribution from NTO3 → NTO4 (Figure S15). Both calculated emission wavelengths (515 and 443 nm) are in good agreement with the experimentally determined maxima (506 and 455, respectively).

## Conclusions

In summary, we have synthesized and characterized physicochemically a series of HfOCs with oxime ligands following a simple one-pot three-component reaction at room temperature. The flexible imide/dioxime ligand H_3_pidiox stabilized a unique pentanuclear {Hf_5_} structure **1**, while the flat catecholate/oxime ligand H_3_dihybo stabilized a hexanuclear {Hf_6_} structure. The {Hf_5_} cluster constitutes the first example of a pentanuclear hafnium(IV) cluster reported to date. The three {Hf_6_} clusters (**2**–**4**) exhibit the same core structure [Hf_6_^IV^(μ_3_-O)_2_(μ_2_-O)_3_] with a trigonal-prismatic arrangement of the six hafnium atoms. Both the core structure and the trigonal-prismatic arrangement of the six hafnium atoms are unique.

The NICS*_zz_* scan curves of the {Hf_6_} system reveal the pronounced metalloaromaticity of the metallic {Hf_3_} ring core. The calculated NICS*_zz_*(1) value of −44.6 ppm is higher than that of −37.3 ppm for benzene and its {Zr_3_} ring core analogue at the same level of theory. The ligation of the catechol oxime ligands to the Hf^IV^ ions, which are in a delocalized co-planar mode, gave rise to a remarkable reduction of the {Hf_6_} clusters’ band gap in comparison to HfO_2_. X-ray diffraction studies and 2D {^1^H} NOESY NMR spectra revealed that the ligands of the two planes defined from each of the two {Hf_3_} cores are at a short distance and interact with each other through π-bonds enhancing further the reduction of the observed band gap. In addition, the pentanuclear and hexanuclear clusters formed by the ligation of the H_3_pidiox and H_3_dihybo to Hf^IV^ result in either emergence (in the case of **1**) or substantial shifting the ligand’s light emission (in the case of **1**–**3**). Theoretical studies revealed that the origin of the luminescence properties observed in {Hf_5_} and {Hf_6_} HfOCs are due to intra-ligand electron transitions. There is a good agreement between the calculated and the experimentally determined emission band maxima. The difference in luminescence activity between the HfOCs and the free ligands might be attributed to the structural features of the former, in which the π orbital manifold of the organic ligands become polarized by their spatial arrangement in the molecules. These findings hint at an alternative strategy to develop new molecule-based materials that are photoactive in the visible region of the electromagnetic spectrum, by employing ligands that are only photoactive with UV light. Furthermore, NMR and ESI-MS studies in solution revealed that the reported HfOCs are thermodynamically stable. The strong chelation of the catecholate–oxime ligand H_3_dihybo to Hf^IV^, and the remarkable aromaticity of the Hf_3_^IV^ rings induce additional stability to the hexanuclear Hf^IV^/H_3_dihybo HfOCs. The facile synthesis, thermodynamic stability, and the unique electronic structure that induces unique properties (aromaticity, fluorescence, low band gap values) to the hexanuclear Hf^IV^/H_3_dihybo HfOCs render them highly promising candidates for the design of novel molecule-based materials with potential applications in chemistry and materials science, such as catalysis, molecular electronic devices, and sensing devices for hafnium. Development of hafnium sensing devices and optoelectronics is currently underway.

## Data Availability

The datasets for the stationary points obtained for the ground and excited state geometries are uploaded in the iochem-bd^87^ database and accessible free of charge via https://doi.org/10.19061/iochem-bd-6-147.

## Supporting Information Available

UV–vis–near-infrared (NIR), Fourier transform infrared (FT-IR), NMR spectra, and crystallographic data (PDF)
