# Supplementary material for: Hafnium(IV) Chemistry with Imide–Dioxime and Catecholate–Oxime Ligands: Unique {Hf5} and Metalloaromatic {Hf6}–Oxo Clusters Exhibiting Fluorescence
Source: Inorg Chem. 2022 Dec 3;61(50):20253–67. doi: 10.1021/acs.inorgchem.2c01768 (PMC9768755; doi:10.1021/acs.inorgchem.2c01768)
Supplement: Supplementary file 1 — ic2c01768_si_001.pdf [file ic2c01768_si_001.pdf]

SUPPORTING INFORMATION

Hafnium(IV) Chemistry with Imide-Dioxime and Catecholate-Oxime Ligands: Unique {Hf_5} and Metalloaromatic {Hf_6}-Oxo Clusters Exhibiting Fluorescence

Stamatis S. Passadis,[a] Sofia Hadjithoma,[b] Nicola J. Fairbairn,[c] Gordon J. Hedley,[c] Nuno A. G. Bandeira,[d]* Athanassios C. Tsipis,[a]* Haralampos N. Miras,[c]* Anastasios D. Keramidas,[b]* and Themistoklis A. Kabanos [a]*

[a]Section of Inorganic and Analytical Chemistry, University of Ioannina, Ioannina 45110, Greece. E-mail: tkampano@uoi.gr

[b]Department of Chemistry, University of Cyprus, Nicosia 1678, Cyprus. E-mail: akeramid@ucy.ac.cy

[c]School of Chemistry, University of Glasgow, Glasgow G12 8QQ, U.K. E-mail: Charalampos.moiras@glasgow.ac.uk

[d]BioISI – BioSystems and Integrative Sciences Institute, Faculdade de Ciências da Universidade de Lisboa, Campo Grande, 1749-016 Lisboa, Portugal. E-mail: nuno.bandeira@ciencias.ulisboa.pt

TABLE OF CONTENTS

1. X-ray Crystallographic Details…..…………………………..………………..…S3

2. X-ray data for compounds **1**, **3** and **4** and Figure S1……………………………S4

3. Structural motifs of HfOCs……………………………………………………..….S5

4. ESI-MS Experimental Details …..…………………………...……………….S7

5. NMR Experimental Details…….………………...………………...……………S7

6. Time resolved fluorescence …………………...……………………………..S8

7. Fluorescence experimental details………………………………………………S8

8. UV-vis studies…………………………………………………………………...S9

9. 2D NMR studies……….…………………………………………….…..…..S11

10. ESI-MS studies……….……………...……………………...…………S12

11. DFT studies…………………………………………………………………S13

12. References……………………………………….……………………..….S17

1. X-ray Crystallographic Details

Suitable single crystals collected and mounted onto a rubber loop using Fomblin oil. Single-crystal X-ray diffraction data of **1** – **3** recorded using an Xcalibur Oxford diffractometer equipped with a Sapphire 3 CCD detector and a 4-cycle Kappa geometry goniometer, using enhanced Mo*Kα* (λ = 0.71073 Å) at 150 K equipped with a graphite monochromator. Analytical absorption correction was applied using CrysAlis RED software. CrysAlis CCD and CrysAlis RED software were used for data collection and data reduction/cell refinement respectively. Structure solution and refinement conducted with SHELXS-2014[1] and SHELXL-2014[2] using the WinGX software package.[3] Corrections for the incident and diffracted beam absorption effects applied using empirical absorption corrections.[4] All the non-H atoms refined anisotropically. Solvent molecule sites found and included in the refinement of the structures. Final unit cell data and refinement statistics for compounds **1**, **3** and **4** are collated in Table S1. The crystallographic data for compounds **1**, **3** and **4** (CCDC 2172158-2172160) can be obtained free of charge from the Cambridge Crystallographic Data Centre, 12, Union Road, Cambridge, CB2 1EZ; fax:(+44)-1223-336-033, deposit@ccdc.cam.ac.uk.

2. Crystallographic data

Table S1. Crystallographic data for compounds **1**, **3** and **4**

	**1**	**3**	**4**
Empirical formula	$C_{43}H_{94}Hf_6KClN_{24}O_{43}$	$C_{50}H_{66}Cl_3Hf_6N_7O_{33}$	$C_{49}H_{61}Cl_2Hf_6N_7O_{61}$
F.W.	2602.43	2470.35	2449.88
T	150(2) K	150(2) K	150(2) K
λ	0.71073 Å	0.71073 Å	0.71073 Å
Crystal system	Tetragonal	Monoclinic	Triclinic
Space group	*P*41	*P*21/c	*P*-1
Unit cell	a = 14.6421(2) Å<br>b = 14.6421(2) Å<br>c = 39.6996(6) Å<br>α = β = γ = 90°	a = 24.6751(3) Å<br>b = 15.1810(2) Å<br>c = 21.3122(3) Å<br>α = γ = 90°<br>β= 113.143(2)°	a = 19.5156(3) Å<br>b = 21.1741(2) Å<br>c = 22.6120(4) Å<br>α= 63.3300(10)°<br>β= 67.168(2)°<br>γ = 84.7750(10)°
V	8511.2(3) $Å^3$	7340.9(2) $Å^3$	7656.2(2) $Å^3$
Z	4	4	4
ρ	1.992 Mg/m^3	2.235 Mg/ m^3	2.125 Mg/ m^3
Absorption coefficient	6.295 mm^{-1}	8.645 mm^{-1}	8.256 mm^{-1}
F(000)	4867	4654	4414
Crystal size(mm^3)	0.090 x 0.070 x 0.040	0.100 x 0.090 x 0.080	0.110 x 0.100 x 0.080
Reflections collected	62548	130219	238063
Independent reflections	25168<br>[R(int) = 0.0317]	55071<br>[R(int) = 0.0232]	110238<br>[R(int) = 0.0222]
Completeness	99.90%	99.70%	99.60%
GoF on F2	1.124	1.023	1.049
Final R indices [I>2sigma(I)]	R1 = 0.0513<br>wR2 = 0.1235	R1 = 0.0359<br>wR2 = 0.0857	R1 = 0.0383<br>wR2 = 0.1024
R indices (all data)	R1 = 0.0619<br>wR2 = 0.1350	R1 = 0.0821<br>wR2 = 0.0934	R1 = 0.0863<br>wR2 = 0.1440

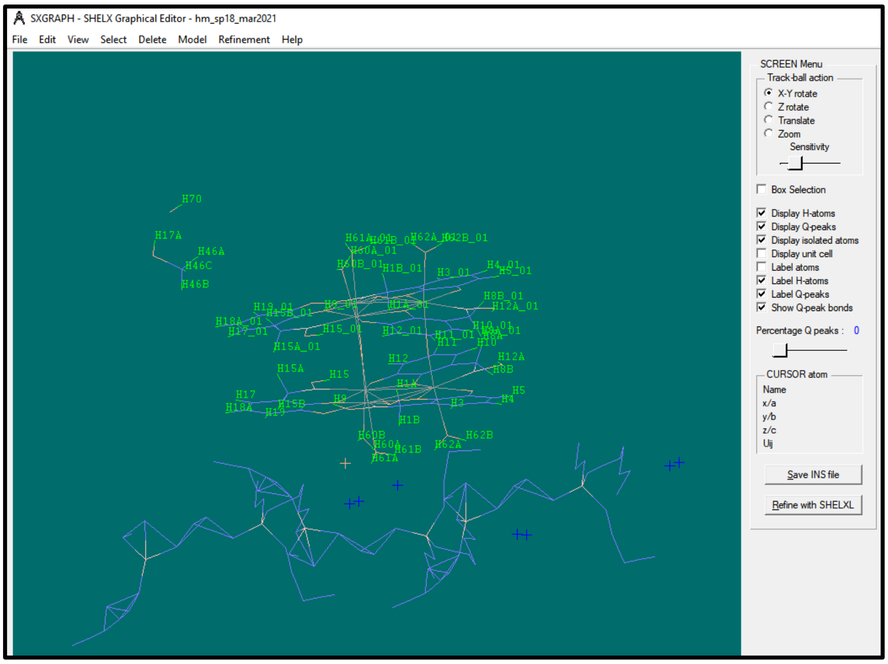

Figure S1. Crystallographic solution of hydroscopic compound **2**. The poor quality of the data didn't allow us to report a structure is publishable form, however allowed us to identify clearly the contents of the unit cell.

3. Structural motifs of HfOCs

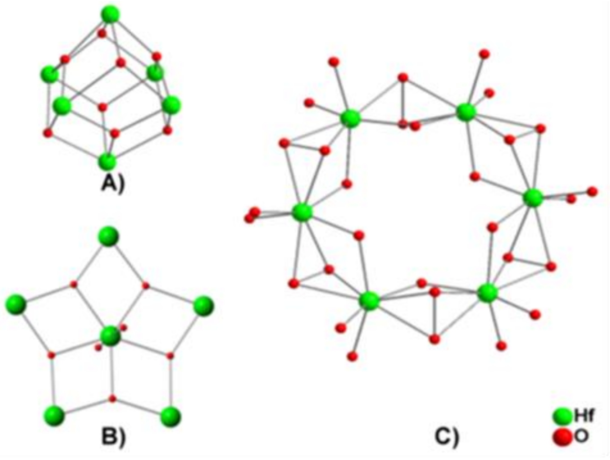

Figure S2. Structural arrangements of the {Hf_6} HfOCs (only the hafnium and bridging oxygen atoms are shown for clarity).

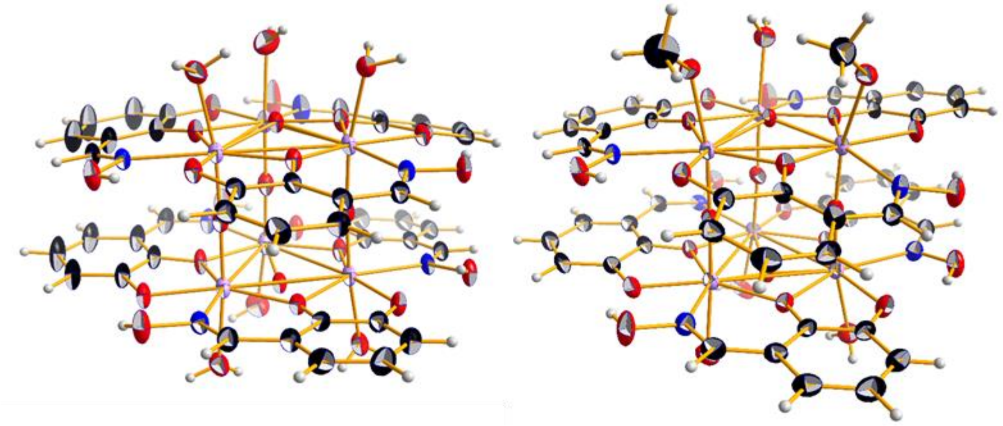

Figure S3. Two {Hf_6} clusters found within the unit cell of HfOC **4** that differ by the two methoxy groups which have replaced the two aqua molecules.

4. ESI-MS Experimental Details

All MS data were collected using a Q-trap, time-of-flight MS (Maxis Impact MS) instrument supplied by Bruker Daltonics Ltd. The detector was a Time-of-Flight, micro-channel plate detector and all data were processed using the Bruker Daltonics Data Analysis 4.1 software, whilst simulated isotope patterns were investigated using Bruker Isotope Pattern software and Molecular Weight Calculator 6.45. The calibration solution used was Agilent ES tuning mix solution, Recorder No. G2421A, enabling calibration between approximately 100 *m/z* and 2000 *m/z*. This solution was diluted 60:1 with MeCN. Samples were dissolved in MeOH and introduced into the MS *via* direct injection at 180 μL h^{-1}. The ion polarity for all MS scans recorded was negative, at 180 ^{o}C, with the voltage of the capillary tip set at 4000 V, endplate offset at –500 V, funnel 1 RF at 300 Vpp and funnel 2 RF at 400 Vpp.

5. NMR Experimental Details

All NMR samples were prepared by the dissolution of the crystals of the HfOCs in CD_3OD at room temperature. ^{1}H, ^{13}C, 2D {^{1}H,^{13}C} *gr*HSQC, 2D {^{1}H,^{13}C} *gr*NOESY NMR spectra were recorded on a Bruker Avance III 300 MHz spectrometer using standard pulse sequences of Bruker Topspin 3.0 software. The 2D {^{1}H} *gr*HSQC spectra were acquired using, 128 increments (with 16 scans each) covering 5.0 ppm at F2 dimension and 150 ppm at F1, and in the case of 2D {^{1}H} *gr*NOESY, the spectra were acquired using, 256 increments (with 16 scans each) covering 5.0 ppm at both F2 and F1. Assignments of the peaks confirmed by spike experiments.

6. Time resolved fluorescence

In both cases we used pulsed laser diode (LDH-D-C-405S, PicoQuant GmbH) as an excitation source at a wavelength 405 nm operating at 40 MHz, with ~ 50 ps pulses (full-width half-maximum). Detection was achieved in a conventional 90° geometry in a 1 cm path length fused silica cuvette. Collected photons were spectrally filtered with a monochromator and then detected on a single photon avalanche photodiode (MPD, PD-100-CTE) that has a transit-time spread of ~ 50 ps (full-width half-maximum). Decays were recorded using a Hydraharp 400 (PicoQuant GmbH) picosecond event timer, operating in a time-correlated single photon counting mode.

7. Fluorescence experimental details

Luminescence spectra were recorded on a Jasco FP-8300 Spectrofluorometer. The relative fluorescence yield of the ligands and the compounds were estimated by comparison to the standard fluorecein (1.00 mM, Φref = 79% in 0.1 M NaOH).[5] Relative fluorescence yields of the compounds were calculated using the following equation.[6-8]

$$\Phi_\chi = (A_{std} / A_x) (F_{std} / F_x) (n_x / n_{std})^2 \Phi_{std} \quad \text{(eq. 1)}$$

where U is the relevant fluorescence yield, A is the absorbance at the excitation wavelength (280 nm), F is the area of the most intense peak of the emission spectrum and is the refractive index of the ‘‘std’’ (standard) and ‘‘x’’ (unknown) solutions (n = 1.329 CH3OH; n = 1.333 H_2O). Measurements of the CH3OH solutions (1.00 x 10^{-3} M) of the crystalline material of the complexes were conducted at room temperature.

8. UV-vis studies

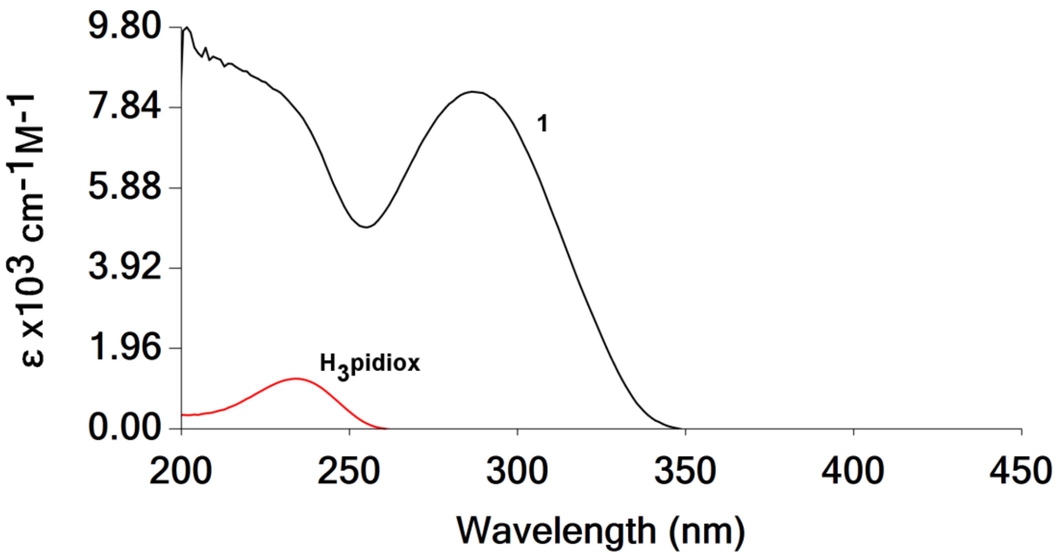

Figure S4. UV-vis spectra of MeOH solutions of H_3pidiox and **1.**

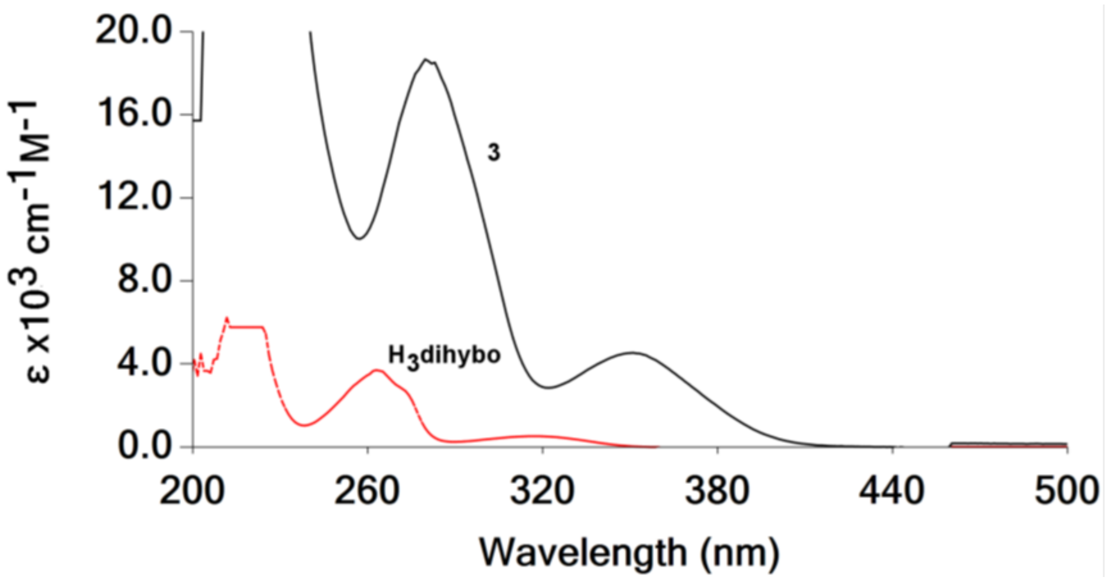

Figure S5. UV-vis spectra of MeOH solutions of H_2dihybo and **3.**

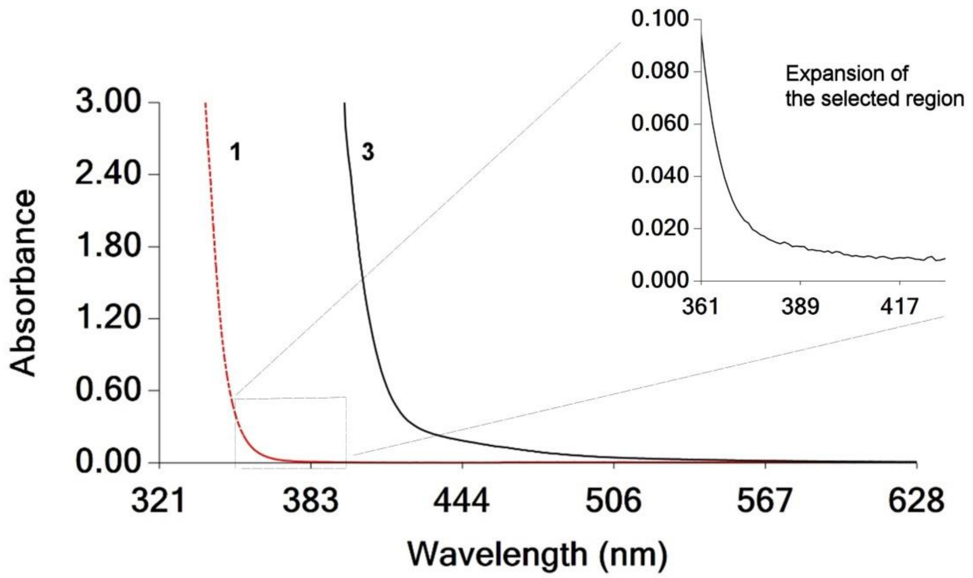

Figure S6. UV-vis spectra of HfOCs **1** and **3** in a concentrated CH_3OH solution (5.0 mM)**.**

8. 2D NMR studies

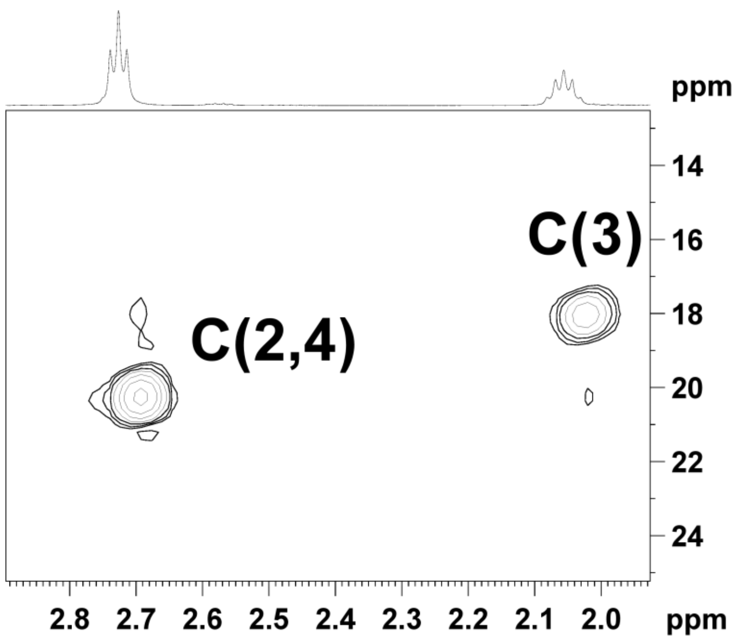

Figure S7. The 2D {^{1}H,^{13}C} grHSQC spectrum of the pentanuclear cluster **1**.

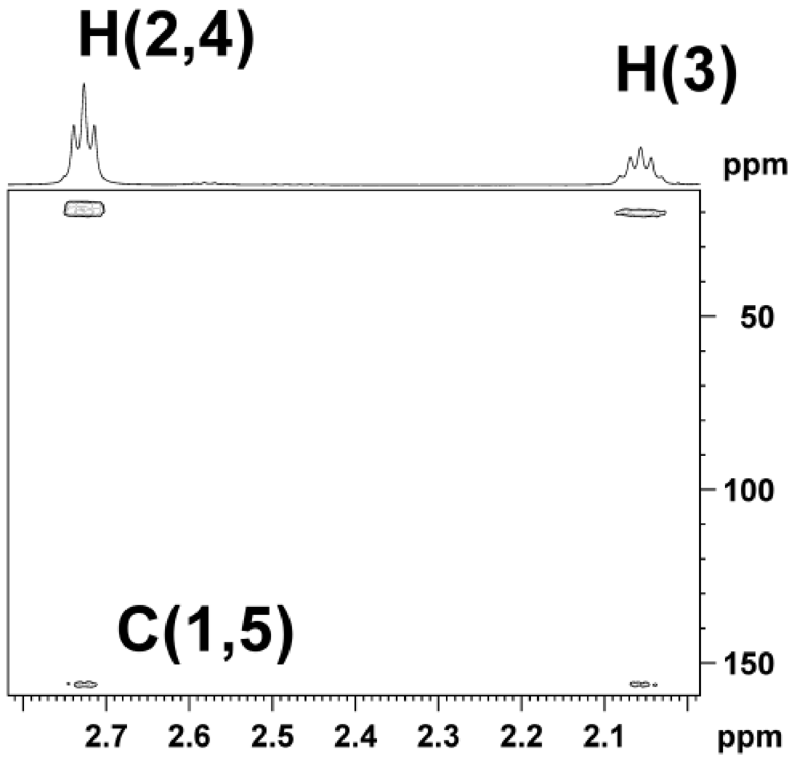

Figure S8. The 2D {^{1}H,^{13}C} grHMBC spectrum of the pentanuclear cluster **1**.

9. ESI-MS studies

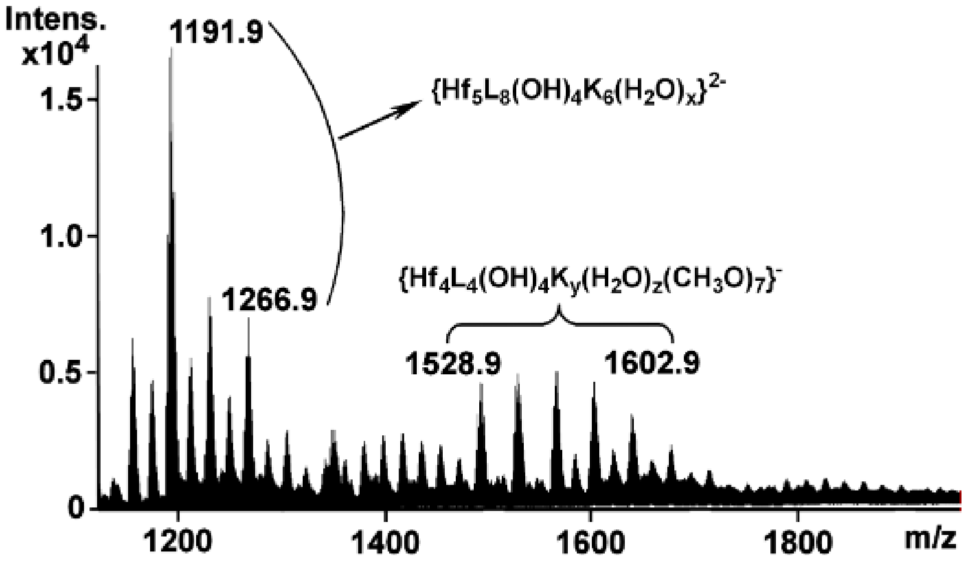

Figure S9. Negative ion mass spectrum of **1** which exhibits two characteristic groups of isotopic distribution envelopes in the range of ca. 1100 - 1300 *m*/*z* with the general formula of $\{Hf_5(C_5N_3O_2H_6)_8(OH)_4K_6(OH_2)_x\}^{2-}$, where x = 0 – 12 and ca. 1500-1600 *m*/*z* with the general formula of $\{Hf_4^{IV}L_4K_y(OH_2)_z(OCH_3)_7\}^{-}$, where y = 0, 1 and z = 0, 2, 3 or 4

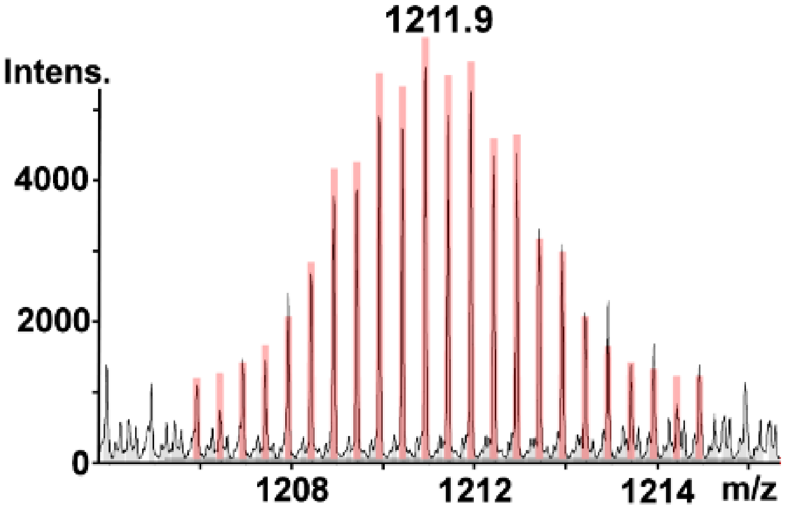

Figure S10. Negative ion mass spectrum in a methanol solution of $\{Hf_5^{IV}(C_5N_3O_2H_6)_8(OH)_4K_6(OH_2)_6\}^{2-}$. Expanded envelope of the doubly charged species centred at *m*/*z ca*. 1211.9. Black line: experimental data, red bars: simulation of isotope pattern.

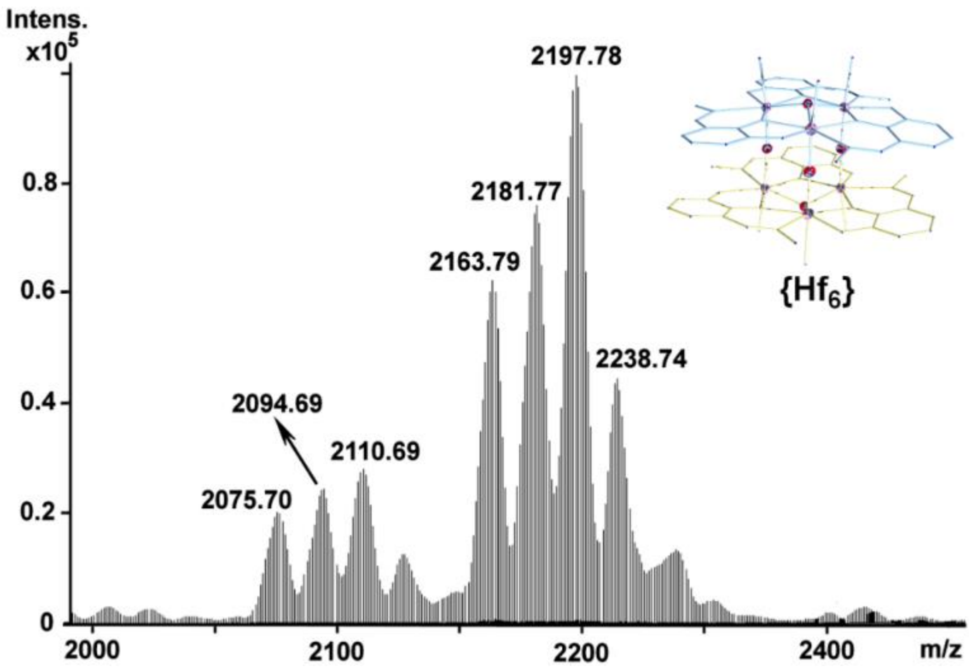

Figure S11. Expanded 2000-2300 *m*/*z* area of the negative ion mass spectrum in a methanol solution of **3**. The observed distribution envelope can be attributed to the intact {Hf_6} species associated with various ratios of solvent molecules and protons. See Table 4 for the assigned species.

10. DFT Studies

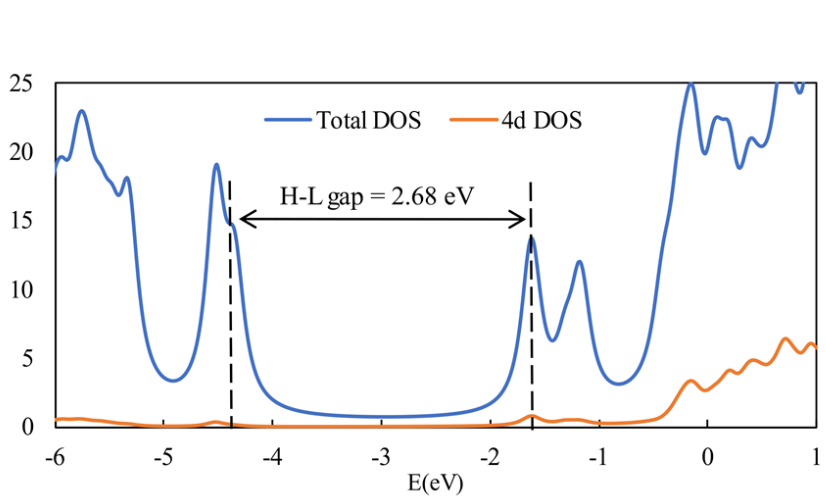

Figure S12. {Hf_5} Total and partial (4d orbital) density of states (DOS) with a Lorentzian broadening of 0.1 eV.

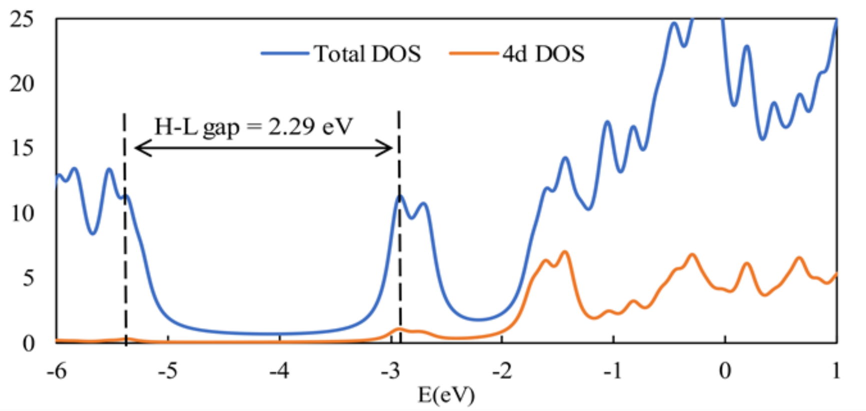

Figure S13. {Hf_6} Total and partial (4d orbital) density of states (DOS) with a Lorentzian broadening of 0.1 eV.

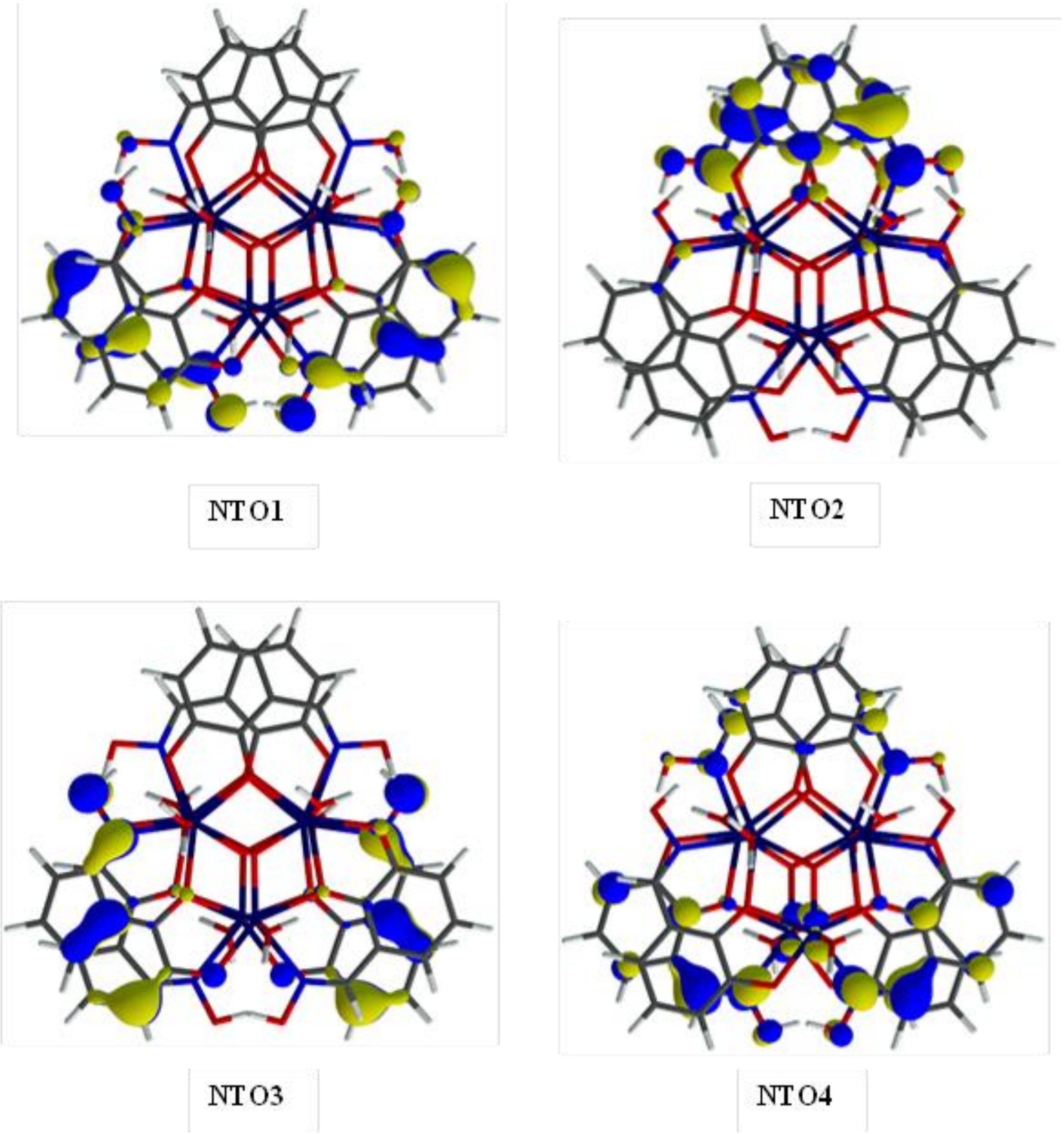

Figure S14. Natural Transition Orbitals pertaining to the emissive state of {Hf_6} (Cf. Table 6).

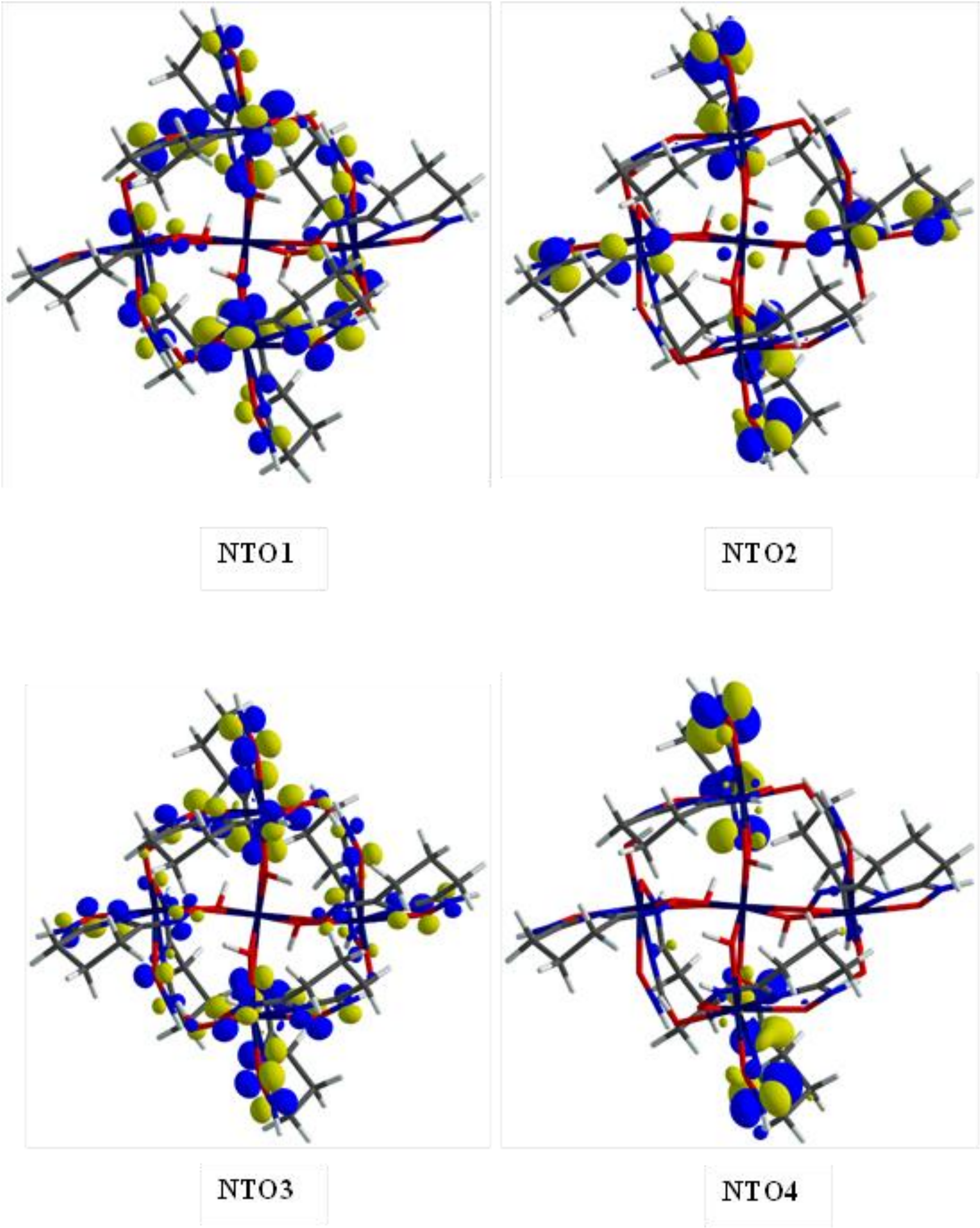

Figure S15. Natural Transition Orbitals pertaining to the emissive state of {Hf_5} (Cf. Table 6).

S12. References

S1. Sheldrick. G. M., Phase annealing in SHELX-90: direct methods for larger structures. *Acta Crystallogr. Sect. A,* **1990**, *46,* 467.

S2. Sheldrick. G. M., A short history of SHELX. *Acta Crystallogr. Sect. A,* **2008**, *64,* 112.

S3. Farrugia. L. J., WinGX suite for small-mo lecule single-crystal crystallography. *J. Appl. Cryst.,* **1999**, *32,* 837.

S4. Clark, R. C.; Reid, J. S., The analytical calculation of absorption in multifaceted crystals. *Acta Crystallogr. Sect. A,* **1995**, *51,* 887.

S5. Umberger, J. Q.; LaMer, V. K. The Kinetics of Diffusion Controlled Molecular and Ionic Reactions in Solution as Determined by Measurements of the Quenching of Fluorescence. *J. Am. Chem. Soc.* **1945**, *67,* (7), 1099-1109.

S6. Panayiotidou, L.; Drouza, C.; Arabatzis, N.; Lianos, P.; Stathatos, E.; Viskadourakis, Z.; Giapintzakis, J.; Keramidas, A. D., Structure, reactivity, luminescence and magnetism of dinuclear Ln 3+ complexes produced by the Ln3+-assisted hydrolysis of 3,6-bis(2-pyridyl)tetrazine. *Polyhedron* **2013,** *64*, 308-320.

S7. Panayiotidou, L.; Stylianou, M.; Arabatzis, N.; Drouza, C.; Lianos, P.; Stathatos, E.; Keramidas, A. D., Synthesis, crystal structure and luminescence of novel Eu3+, Sm3+ and Gd3+ complexes of 1,3,5- and 1,2,4-triazines. *Polyhedron* **2013,** *52*, 856-865.

S8. Latva, M.; Takalob, H.; Mukkala, V. M.; Matachescu, C.; Rodríguez-Ubis, J. C.; Kankare, J., Correlation between the lowest triplet state energy level of the ligand and lanthanide(III) luminescence quantum yield. *J. Lumin.* **1997,** *75* (2), 149-169.
